# Mesoporous Carbons and Highly Cross-Linking Polymers for Removal of Cationic Dyes from Aqueous Solutions—Studies on Adsorption Equilibrium and Kinetics

**DOI:** 10.3390/ma17061374

**Published:** 2024-03-17

**Authors:** Malgorzata Zienkiewicz-Strzalka, Magdalena Blachnio, Anna Derylo-Marczewska, Szymon Winter, Malgorzata Maciejewska

**Affiliations:** Faculty of Chemistry, Maria Curie-Sklodowska University, M. Curie-Sklodowska Sq. 3, 20-031 Lublin, Poland; malgorzata.zienkiewicz-strzalka@mail.umcs.pl (M.Z.-S.); magdalena.blachnio@mail.umcs.pl (M.B.); szymonwinter123@wp.pl (S.W.); mmacieje@umcs.pl (M.M.)

**Keywords:** mesoporous carbon, mesoporous polymer, hierarchical porosity, dye adsorption, methylene blue, crystal violet, malachite green, nanostructure, environment protection

## Abstract

This study presents the results of applying the methods of synthesizing mesoporous carbon and mesoporous polymer materials with an extended porous mesostructure as adsorbents for cationic dye molecules. Both types of adsorbents are synthetic materials. The aim of the presented research was the preparation, characterisation, and utilisation of obtained mesoporous adsorbents. The physicochemical properties, morphology, and porous structure characteristics of the obtained materials were determined using low-temperature nitrogen sorption isotherms, X-ray diffraction (XRD), small angle X-ray scattering (SAXS), and potentiometric titration measurements. The morphology and microstructure were imaged using scanning electron microscopy (SEM). The chemical characterisation of the surface chemistry of the adsorbents, which provides information about the surface-active groups, the elemental composition, and the electronic state of the elements, was carried out using X-ray photoelectron spectroscopy (XPS). The adsorption properties of the mesoporous materials were determined using equilibrium and kinetic adsorption experiments for three selected cationic dyes (derivatives of thiazine (methylene blue) and triarylmethane (malachite green and crystal violet)). The adsorption capacity was analysed to the nanostructural and surface properties of used materials. The Generalized Langmuir equation was applied for the analysis of adsorption isotherm data. The adsorption study showed that the carbon materials have a higher sorption capacity for both methylene blue and crystal violet, e.g., 0.88–1.01 mmol/g and 0.33–0.44 mmol/g, respectively, compared to the polymer materials (e.g., 0.038–0.044 mmol/g and 0.038–0.050 mmol/g, respectively). The kinetics of dyes adsorption was closely correlated with the structural properties of the adsorbents. The kinetic data were analysed using various equations: first-order (FOE), second-order (SOE), mixed 1,2-order (MOE), multi-exponential (m-exp), and fractal-like MOE (f-MOE).

## 1. Introduction

Structures based on porous carbons form an important group of nanomaterials and functional nanocomposites intended for natural environment protection including protection against pollution [1,2,3,4] and greenhouse gas emissions [5,6]. They are used in low-emission systems and as strategic substitutes for environmentally hazardous materials [7,8,9]. Porous carbons are known for their environmental efficiency and high recyclability. Finally, they enable the development of innovative technologies for producing new and functional adsorbents, elements of electrochemical devices and energy storage, electrode materials catalyst supports, bioscaffolds, and biosensors [10,11,12,13,14,15,16,17].

The family of nanostructured carbon forms is very rich and has been developed over the last 20 years [18,19,20,21]. It includes activated carbons, ordered porous carbons, mesoporous and microporous carbon materials, and hierarchical porous carbon forms. Activated carbon is produced by heating carbon-rich materials such as coconut shells [22,23] or wood [24,25,26,27] in the presence of a gas, creating many small pores. Activated carbon with well-developed microporosity is known for its large surface area, which makes it useful for applications such as adsorption, separation, and environmental remediation; however, its applicability may be limited for substances with large molecular sizes. Ordered porous carbon has a well-defined and ordered pore structure, usually created through the templating during their synthesis. Ordered porous carbons are often appreciated in catalysis and gas separation [18,28]. However special interest is caused by the mesoporous forms, which have medium-sized pores, typically in the range of 2 to 50 nm. Mesoporous ordered carbons are used in drug delivery and catalysis [29,30,31,32,33,34,35,36,37]. Finally, hierarchical porous carbons are a type of carbon material that have a complex pore structure with different pore sizes and distributions [38,39,40,41,42,43]. These materials are typically synthesized through a combination of templating and carbonisation processes, which generate the hierarchical pore structure. A large surface area and open access network make them useful for energy storage, catalysis, and gas separation. In addition, their porous nature allows for easy diffusion of molecules, making them ideal for adsorption and separation processes [44,45,46,47,48].

A variety of approaches have been developed for the production of carbon materials with enhanced porosity. These include a hard templating method, a soft templating method, and a combined templating/activation method. The hard templating method is a popular approach for synthesizing carbon materials with hierarchical porosity. In this method, a sacrificial template material such as silica, silica nanophases [40,49], or insoluble metal, metal oxide, and metal hydroxide templates such as alumina allow porosity control. In the soft templating method, a self-organized block copolymer, consisting of two or more chemically different polymers spontaneously organizing into a well-defined periodic structure, is used as a template. The resulting carbon structure inherits the hierarchical porosity from the template, with the pore sizes and distributions determined by the type of the block copolymer. The advantage of this method is that it allows the synthesis of carbon materials with a wide range of pore sizes and shapes. In the combined template/activation method, a suitable template is used to create a carbon structure, and an activation process is used to modify its properties. The template can be made from a variety of materials such as silicon dioxide or polymers and is used to create a specific carbon structure. The activation process can involve chemical or physical treatments, such as heating or etching, which change the carbon structure and create new surface sites. A great advantage of mesoporous carbon materials is the possibility of their easy recycling and effective reuse. In this way, the costs of producing new materials and the amounts of the deposits of spent adsorbents can be reduced. Such action is also part of sustainable material management. Regarding the regeneration of activated carbons with adsorbed dyes, the commonly used method is based on the desorption of the adsorbate using various agents such as bases, acids, oxidants, organic solvents, water, and salts [50,51]. Moreover, physical regeneration by thermal treatment under an inert atmosphere or using microwaves is also widely applied [52,53].

Another valuable class of porous materials is highly cross-linked organic polymers. Generally, they are obtained via radical copolymerisation with the presence of diluents. In the course of polymerisation, at a certain degree of conversion, the process of phase separation occurs. The initially homogeneous polymerisation mixture is split into solid and liquid phases. The solid phase is composed of a cross-linked polymer network, whereas the solvent and unreacted comonomers make up the liquid phase. The phase separation is induced by the increase of cross-linking degree (*ν*-syneresis) or by the change in the polymer–solvent interaction (macro- or microsynersis). In microsyneresis, the liquid phase is dispersed in the polymer gel phase whereas in macrosyneresia, nuclei are formed. The nuclei are microgels with a higher-than-average cross-linking density surrounded by a liquid phase. Nuclei react with each other through free double bonds, forming macrogels. The free space between macrogels generates mesopores whereas micropores are present between nuclei. The occurrence of macrogel agglomerates leads to macropores. The phase separation, and consequently the polymer porosity, depends on multiple polymerisation variables; e.g., the type of cross-linking monomer, the porogenic solvent (type and amount), and the ratio between the functional and the cross-linking monomers. By adjusting these independent parameters, diverse porous structures can be obtained [54,55,56,57,58]. After using highly cross-linked organic polymers as adsorbents, they can be regenerated. As the copolymers are stable in the whole range of pH, various desorbing agents can be applied. Additionally, the good thermal resistance of the investigated copolymers enables drying at elevated temperatures. The combination of both thermal and chemical regeneration techniques is also a promising option.

In this work, the adsorption properties of mesoporous carbon and polymer materials are investigated. The synthesis methods allow the design of adsorbents with divergent porosity and physicochemical characteristics by changing the reaction mixture composition and the conditions in which the process is conducted. As a result, the materials of differentiated surface and structural properties were obtained which show a different affinity towards selected dyes. Research on the relationship between the type of porosity and chemical composition of the adsorbent surface, and the uptake and rate of adsorption, is the basis for designing adsorbents for specific applications. To synthesise carbon materials with extended and hierarchical porosity, the amphiphilic triblock copolymers with the basic molecular structure polyoxyethylene–polyoxypropylene–polyoxyethylene (PEO–PPO–PEO), (PE6400 and PE9400), were used as nanostructure directing agents of the mesoporous silica phase and a source of the carbon phase during carbonisation. For the synthesis of the porous copolymers, a suspension copolymerisation was used. The adsorption properties of the obtained materials with developed mesoporosity were studied for the adsorbates characterized by larger molecular sizes for which the microporous activated carbons are not useful as effective adsorbents. The equilibrium and kinetic measurements were conducted to determine the adsorbents’ effectiveness, taking into account the adsorption rate and capacity. The adsorption capacity and rate were correlated with the adsorbate properties, and the surface and structural characteristics of synthesized materials.

## 2. Materials and Methods

### 2.1. Chemicals

Pluronic PE9400 and PE6400 copolymers were purchased from BASF (West Port Arthur Road, Beaumont, TX, USA). Tetraethyl orthosilicate (TEOS), phenyltriethoxysilane (PhTEOS), and 1.3.5-trimethylbenzene (TMB) were supplied by Sigma-Aldrich (Poznan, Poland). Toluene, dodecane, acetone, methanol (reagent grade), HCl, and sulphuric acid H_2_SO_4_ (concentration 95%) were purchased from POCh (Gliwice, Poland). Divinylbenzene (DVB), (purchased from Merck, Darmstadt, Germany), trimethylpropane trimethacrylate (TRIM) (from Fluka, Buchs, Switzerland), and 1-vinyl-2-pyrrolidone (VP) (from Fluka, Buchs, Switzerland) were washed with 5% aqueous sodium hydroxide to remove inhibitors. Poly(vinyl alcohol) and α,α’-azoisobisbutyronitrile from Fluka, Buchs, Switzerland were used without purification. The methylene blue (MB), malachite green (MG), and crystal violet (CV) were supplied by Sigma-Aldrich (Poznan, Poland) or POCh (Gliwice, Poland).

### 2.2. Adsorbents Preparation

#### 2.2.1. Synthesis of Mesoporous Carbons

**Figure 1:**
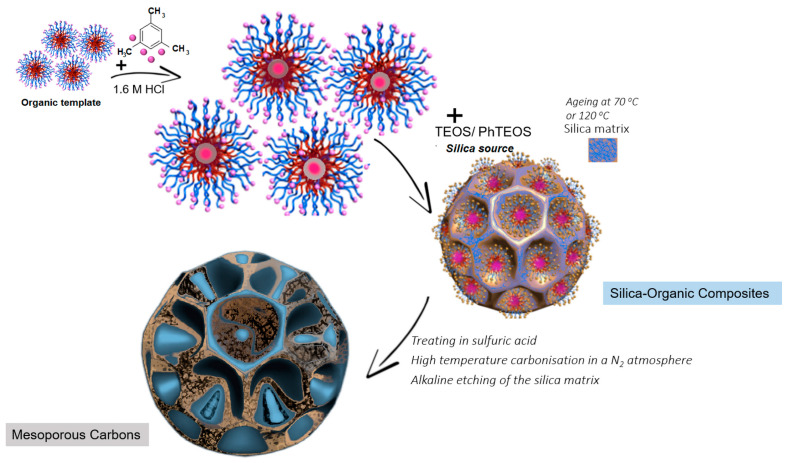
Scheme for the synthesis of hierarchical (mesoporous) porous carbon materials via a templating-free approach.

The synthesis of mesoporous carbons was based on the direct synthesis method described in the literature [59], to which some modifications were made. In the first stage, silica–organic composites were prepared using the following reagents: (i) non-ionic Pluronic-type triblock copolymers; (ii) tetraethyl orthosilicate (TEOS) and phenyltriethoxysilane (PhTEOS) as precursors of the silica phase; (iii) 1,3,5-trimethylbenzene (TMB) as an expander; and (iv) 1.6 M HCl solution as a reaction medium. The initial relative amounts of the synthesis reagents were as follows: the concentration of copolymer was fixed at 2.7%, the mass ratio of polymer to expander was maintained as 1:1, the mass ratio of copolymer to TEOS and PhTEOS was 1.7:4.7:1. To obtain different silica–organic composites, two Pluronic copolymers with different physicochemical properties, i.e., PE9400 and PE6400, and two aging temperatures of the material in the mother solution, i.e., 70 °C and 120 °C, were used. In the second stage, the silica–organic composites were treated with the addition of sulfuric acid as a catalyst: (i) vacuum drying at two temperatures; (ii) high-temperature carbonisation in a nitrogen atmosphere; and (iii) alkaline etching of the silica matrix. The copolymer reagents and parameters used in the synthesis of mesoporous carbon are listed in Table 1. In the proposed direct synthesis method, the organic matrix serves both as a blowing agent in the primary silica material and as a carbon precursor. For this reason, the calcination process of the silica–organic composite is omitted here, which is fundamentally different from the hard templating method in which the silica matrix is impregnated with a substance that is a source of organic carbon (e.g., sugar [60]). In Figure 1, the scheme for the synthesis of hierarchical (mesoporous) porous carbon materials via a templating-free approach is presented.

#### 2.2.2. Synthesis of Mesoporous Polymer Materials

**Figure 2:**
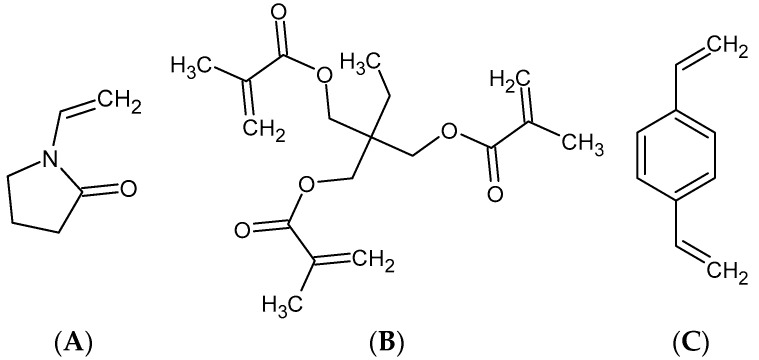
Chemical structures of used monomers:1-vinyl-2-pyrrolidone (**A**), trimethylpropane trimethacrylate (**B**), divinylbenzene (**C**).

The mesoporous polymer materials were obtained by a suspension copolymerisation approach using equivalent mole fractions of functional monomer 1-vinyl-2-pyrrolidone (VP) and cross-linkers (divinylbenzene (DVB) or trimethylpropane trimethacrylate (TRIM)) (Figure 2). The copolymerisation processes were carried out in an aqueous suspension medium. In the course of the experiment, 195 mL of distilled water and 6.5 g of poly(vinyl alcohol) (PVA) were stirred for 6 h at 80 °C in a three-neck flask. Additionally, in the case of synthesis P2 copolymer, 30 g of CaCl_2_ was added to the aqueous medium. Then, the solution was prepared with 15 g of monomers and 0.075 g of α,α’-azoisobisbutyronitrile in 22.5 mL of diluent (toluene + n-dodecane) and added to the aqueous medium. The copolymerisation was carried out for 20 h at 80 °C. The resulting porous beads were aspirated, washed with hot water, and extracted in a Soxhlet apparatus with acetone, toluene, and methanol. Experimental details of the process and amount of the components are summarized in Table 2.

### 2.3. Adsorbate Molecules

**Figure 3:**
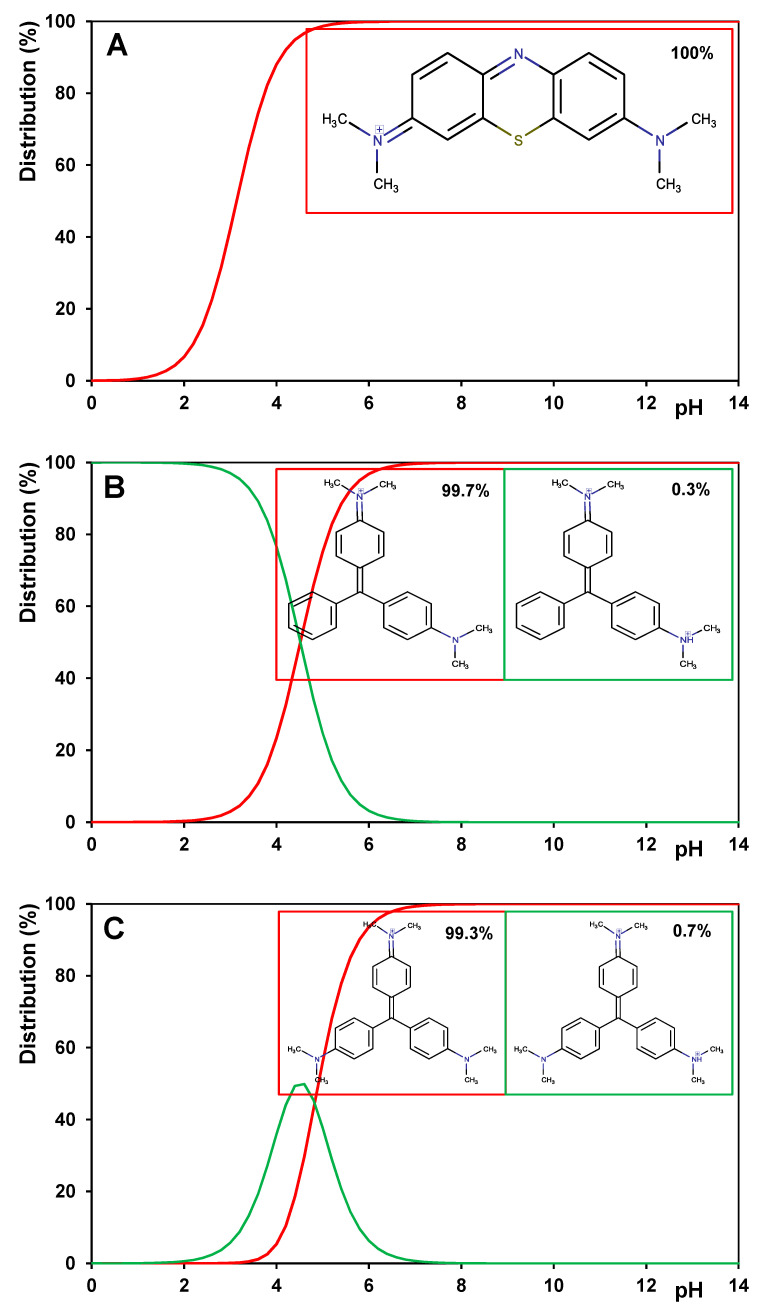
Percentage of the molecular forms of adsorbates under experimental pH (pH = 7) for (**A**) methylene blue, (**B**) malachite green, and (**C**) crystal violet.

Three cationic dyes were used in adsorption from the liquid phase. They constitute derivatives of thiazine (methylene blue; MB) and triarylmethane (malachite green; MG and crystal violet; CV). The structure and physicochemical properties of the adsorbates are summarised in Table 3. The chemical structures of given molecules with their maximal size are shown in Figure S1 in Supplementary Materials. Moreover, the distribution of the molecular forms of the adsorbates under an experimental pH (pH = 7) is presented in Figure 3.

### 2.4. Methods of Investigations

#### 2.4.1. Nitrogen Adsorption/Desorption Measurement

The textural characterisation and evaluation of the porosity of the mesoporous carbon and polymer samples was performed by isothermal nitrogen adsorption–desorption measurements at a low temperature using an ASAP2020 instrument (Micromeritics, Norcross, GA, USA). The specific surface areas were calculated from the experimental isotherms using BET theory and the linear region of the BET plot. The pore size distributions were calculated according to the method of Barrett, Joyner, and Halenda (BJH). All samples were degassed for 24 h at 100 °C in the degassing port of the analyser before the adsorption/desorption analysis.

#### 2.4.2. X-ray Diffraction (XRD) and Small Angle X-ray Scattering (SAXS)

The synthesized materials were investigated by the XRD technique using a PANalytical Empyrean (PANalytical, 2012, Malvern, UK) diffractometer system equipped with a sample spinner as interchangeable X-ray modules. X-ray tubes with Cu anode materials were utilised as radiation sources (X-ray wavelength 1.5418 Å). As a detector, the PIXcel3D was used in linear (1D) scanning mode. The incident beam path consisted of W/Si, a stepped X-ray mirror with an elliptical shape. Scans were made over a 2θ range of 5–90° with a total exposure time of 1 h. SAXS analysis was performed using the same device, an Empyrean diffractometer (PANalytical, 2012, UK) with SAXS/WAXS sample stage with capillary mode. The device was operated with a generator setting of 40 kV and 40 mA. The SAXS measurements were performed at 0.1–4° 2θ with a step size of 0.005. The primary beam was measured with a Cu 0.2 mm beam attenuator and a PIXcel3D detector. The length of the scattering vector (or scattering vector) q is defined as q = (4πsinθ)/λ, where 2θ is the scattering angle and λ is the X-ray wavelength (1.5418 Å). Background correction was performed by an air scattering measurement using an empty sample holder with protected foil before sample measurement. The SAXS calculations were performed with the EasySAXS software (PANalytical, UK, version 1.2).

#### 2.4.3. Scanning Electron Microscopy

The scanning electron microscopic (SEM) analysis was performed with the QuantaTM 3DFEG (FEI Company, Hillsboro, OR, USA) device at 5 kV. A high vacuum (4 × 10^−4^ Pa) was used to image the analysed hydrogel samples. Before the SEM measurements, the samples were coated with gold to improve their electrical conductivity and their ability to reflect electrons, and thus provide clear images.

#### 2.4.4. X-ray Photoelectron Spectroscopy

X-ray photoelectron spectroscopy data were collected on the Multi-chamber UHV System, Prevac (2009, Rogow, Poland) using the hemispherical analyser ScientaR4000 by monochromatic Al Ka radiation from high-intensity source MX-650, Scienta (Uppsala, Sweden). High-resolution XPS data were obtained for O1s and C1s signals.

#### 2.4.5. Potentiometric Titration Measurement

The surface charge densities and the values of the zero point of charge (pHpzc) of the adsorbents were determined using the potentiometric titration method. The titration set consisted of an automatic burette Dosimat 765 (Metrohm, Herisau, Switzerland), a pHmeter (PHM 240, Radiometer, Copenhagen, Denmark), and a thermostatic quartz vessel. After acidification (with HCl solution) of the suspension (adsorbent + electrolyte solution), titration with NaOH solution and pH measurements were performed. To exclude errors associated with pH changes due to the dissolution of carbon dioxide in the suspension solution, the experiments were carried out in a nitrogen atmosphere.

#### 2.4.6. Adsorption Studies

Adsorption isotherms of dyes from aqueous solutions were determined using a static method, but adsorption experiments were performed differently depending on the type of adsorbent. In the case of carbon materials, the weighted amount of adsorbent (~0.02 g) was contacted with 5 mL of water and degassed under a vacuum. An adsorbent was then poured over with a dye solution (pH = 7), placed in an incubator shaker at 25 °C (Innova 40R, New Brunswick Scientific, Edison, NJ, USA), and kept there until adsorption equilibrium was reached. A sample of the dye solution was then taken, and its concentration was determined by using the Cary 100 UV/Vis spectrophotometer (Varian Inc., Melbourne, VIC, Australia). Then, a certain amount of the dye stock solution was added to an adsorption system. These activities were repeated several times to measure the adsorption isotherms over a wide concentration range (0.07–0.92 mmol/L for MB; 0.07–0.55 mmol/L for MG; 0.05–0.32 mmol/L for CV). For polymeric materials, the series of adsorbent samples (~0.1 g for MB; ~0.08 g for CV) were contacted with water, degassed, and poured with dye solutions of different concentrations (0.02–0.42 mmol/L for MB; 0.01–0.20 mmol/L for CV) and placed in an incubator shaker at 25 °C. After attaining adsorption equilibrium, the solute concentrations were determined based on the spectrophotometric measurements.

In order to keep the pH conditions of the adsorption experiments constant, solutions of all dyes were prepared using a buffer with pH = 7.

The analysis of the equilibrium data was based on the Generalized Langmuir equation (GL) [62,63,64]:

(1) $$\theta ={\left[\frac{{\left(Kc_{eq}\right)}^{n}}{1+{\left(Kc_{eq}\right)}^{n}}\right]}^{\frac{m}{n}}$$

where *θ* = a_eq_/a_m_ is the global adsorption isotherm; a_m_ is the adsorption capacity; m, n are the heterogeneity parameters; and K is the equilibrium constant.

Depending on the specific values of *m* and *n* parameters, the GL equation is simplified into four equations: Langmuir (L) (GL: *m* = *n* = 1), Langmuir–Freundlich (LF) (GL: 0 < *m* = *n* ≤ 1), Generalized Freundlich (GF) (GL: *n* = 1, 0 < *m* ≤ 1), and Tóth (T) (GL: *m* = 1, 0 < *n* ≤ 1). The Generalized Langmuir isotherm (GL) is usually applied for the analysis of localised physical adsorption on energetically heterogeneous solids and despite its relatively simple form, it describes most of the available experimental data quite well. The GL equation corresponds to an asymmetric quasi-Gaussian adsorption energy distribution function; the heterogeneity parameters m and n characterise the extension of this function towards higher (m < 1) and lower (n < 1) adsorption energies (the smaller m and n is, the greater the heterogeneity). The Tóth equation corresponds to an asymmetric quasi-Gaussian adsorption energy distribution function extended towards low energies, while the GF isotherm corresponds to an exponential function with a characteristic minimum energy. The Langmuir–Freundlich (LF) equation conforms to a symmetric quasi-Gaussian adsorption energy distribution function, and equation (L) describes adsorption on a solid characterized by energetic homogeneity.

Similar to the adsorption equilibrium experiments, the kinetic measurements were also carried out using a UV/Vis spectrophotometer. The dye solution of a certain concentration was brought into contact with the known amount of solid in a glass vessel. During the entire experiment, the suspension solution was stirred magnetically, and at a certain point in time, the solution samples were collected in a measuring cell and measured. The recorded spectra were used to determine the experimental profiles of the concentration as a function of time, which were then analysed using the various kinetic equations: the first-order kinetic equation (FOE), the second-order equation (SOE), the mixed 1,2-order equation (MOE), the fractal-like MOE equation (f-MOE), and the multi-exponential equation (m-exp). General and/or linear forms of the specific equations, along with their half-time expressions are as follows:

- First-order equation [65,66]
  (2) $$\frac{dc}{dt}={-k}_{1}(c-c_{eq})$$
  (3) $$\ln\left(\;c-c_{eq}\right)=\ln\left(c_{o}-c_{eq}\right)-k_{1}t$$
  (4) $$t_{0.5}\sim 1/k1$$
  where *c* is the temporary concentration, the *c_o_* and *c_eq_* are initial and equilibrium concentrations, *k*_1_ is the adsorption rate coefficient, *t* is time;
- Second-order equation [65]
  (5) $$\frac{dc}{dt}={-k}_{2c}{\left(c-c_{eq}\right)}^{2}$$
  (6) $$t_{0.5}\sim 1/k2$$
- 1,2-mixed-order equation [65]
  (7) $$F=a/a_{eq}=\frac{1-exp\left(-k_{1}t\right)}{1-f_{2}exp\left(-k_{1}t\right)}$$
  (8) $$ln\left(\frac{1-F}{1-f_{2}F}\right)=-k_{1}t$$
  (9) $$t_{0.5}\sim \mathrm{1n}(2\;-\;f_{2})]/k_{1}$$
  where *F* is the relative adsorption progress in time, *f_2_ <* 1 is the normalized share of the second-order process in the kinetics. In special cases, the MOE equation may be degraded to the simple kinetic equations of the first- (*f_2_ =* 0) and the second-order (*f_2_ =* 1) type;
- Multi-exponential equation [62,65,66]
  (10) $$c=\left(c_{o}-c_{eq}\right)\sum_{i=1}^{n}f_{i}exp\left(-k_{i}t\right)+c_{eq}$$
  (11) $$t_{0.5,i}\sim (\ln\;2)/ki$$
  where “*i*” is the term of m-exp equation, *k_i_* is the rate coefficient, and u_eq_ = 1 − c_eq_/c_0_ is the relative loss of adsorbate from the solution;
- Fractal-like MOE equation [65,66]
  (12) $$F=\frac{{1-exp\left(-k_{1}t\right)}^{p}}{{1-f_{2}exp\left(-k_{1}t\right)}^{p}}$$
  (13) $$ln\left(\frac{1-F}{1-f_{2}F}\right)=-(k_{1}{t)}^{p}$$
  (14) $$t_{0.5}\sim {\left[\ln(2\;-\;f_{2})\right]}^{1/p}/k1$$
  where *p* is the fractal parameter.

## 3. Results and Discussion

### 3.1. Adsorbents Characteristics

#### 3.1.1. Porosity Characteristics by Low-Temperature N_2_ Adsorption/Desorption

**Figure 4:**
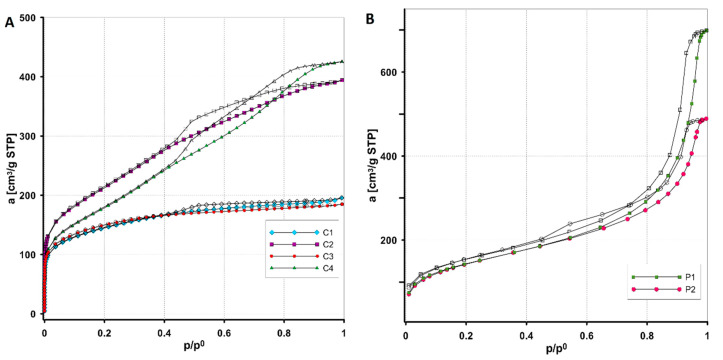
Nitrogen adsorption/desorption isotherms on activated carbons (**A**) and polymers (**B**). Solid symbols indicate the adsorption branch, empty symbols indicate the desorption branch.

**Figure 5:**
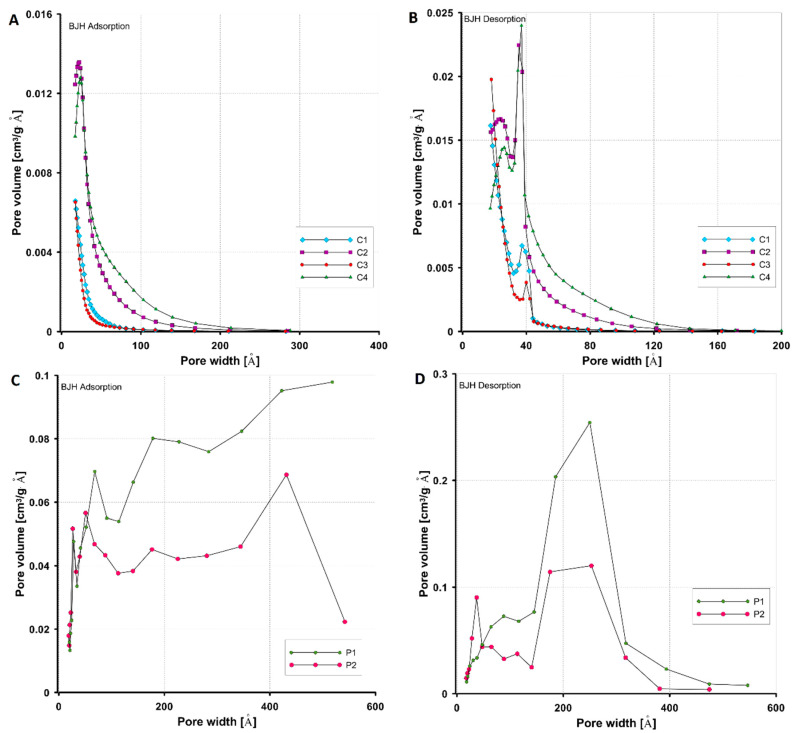
Comparison of pore volume distributions in relation to their sizes determined from the adsorption and desorption branches of gas isotherms for activated carbons (**A**,**B**) and polymers (**C**,**D**).

The structural properties of the synthesized adsorbents (C1–C4 and P1, P2) were determined based on standard measurement methods of low-temperature nitrogen adsorption/desorption isotherms. Figure 4 and Figure 5 show the measured isotherms and the pore distributions calculated with the Barrett–Joyner–Halenda (BJH) method for all tested materials. The analysis of nitrogen adsorption isotherms for mesoporous carbon materials (C1–C4) (Figure 4A) revealed two types of adsorbent structures, namely a mixed type with the participation of micro- and mesopores (the carbons C1 and C3) and a borderline type characterized by the presence of almost exclusively mesopores (C2 and C4). According to the IUPAC classification, the isotherms are hybrids of type I and type IV with a hysteresis loop of type H4 (the carbons C1 and C3) and the typical type IV with a hysteresis loop of type H3 (the carbons C2 and C4). Compared to the others, the carbons C2 and C4 have a better-developed porous structure and a larger pore volume, which is reflected in much higher adsorption values at a relative pressure close to unity. In addition, adsorption on these materials systematically increases with increasing pressure, indicating a significant change in pore size in which subsequent batches of adsorbate condense. The types of hysteresis loops observed in the course of the adsorption and desorption curves indicate that the carbons are characterized by the presence of pores in the form of slits (the carbons C2 and C4) or narrow slits (the carbons C1 and C3) formed between two planes.

The nitrogen adsorption/desorption isotherms on the polymeric materials (Figure 4B) show a similar pattern, although the polymer P1 exhibits higher adsorption, especially in the high relative pressure region. The mesoporous nature of the tested polymers is evidenced by the presence of a well-defined hysteresis loop in the nitrogen adsorption/desorption isotherms. It should also be noted that for these materials at relatively high relative pressures (p/p_0_ > 0.8), a significant increase in adsorption corresponding to condensation in mesopores is observed, indicating the presence of large pores. The isotherms for both polymers belong to type IV with a hysteresis loop of type H3 (slotted pore shape).

Analysing the pore size distribution functions for the carbon materials (Figure 5A,B), it is observed that they represent a bimodal type with two significant maximum pore diameters. The distributions corresponding to the carbons C2 and C4 are broader at the base in terms of larger values of pore diameters compared to the others, indicating some variation in the sizes of the mesopores. Higher maximum values on the difference curves indicate that these carbons have a larger pore volume. The pore distribution functions for polymeric materials determined from the desorption branch of the isotherm (Figure 5C,D) are of the homogeneous (Gaussian) type (polymer P1) and the bimodal type (polymer P2). For both materials, the main peak is located in the region of large pore diameters (mesopore), while in the case of the polymer P2 there is also a population of smaller mesopores. Table 4 shows the values of the parameters characterizing the porous structure of the adsorbents, i.e., the specific BET surface area (S_BET_), the total pore volume (V_t_), the micropore volume (V_mic_), the average hydraulic pore diameter (D_h_), the average BJH adsorption pore diameter (D_BJH ads_), and the average BJH desorption pore diameter (D_BJH des_). As the data presented show, the carbons C2 and C4 have the largest specific surface area (760 m^2^/g and 649 m^2^/g). The carbons C1 and C3 have the smallest specific surface area (502 m^2^/g and 508 m^2^/g), and also have the smallest total pore volume (0.29–0.3 cm^3^/g). The polymeric materials have the same specific surface area (515 m^2^/g), but a different total pore volume (1.06 cm^3^/g and 0.75 cm^3^/g) and a different hydraulic pore size (8.3 nm and 5.8 nm). The V_t_ and D_h_ values for polymers are the highest compared to those for other materials.

#### 3.1.2. Structure of Nano-Sized Materials by XRD and Small-Angle X-ray Scattering

**Figure 6:**
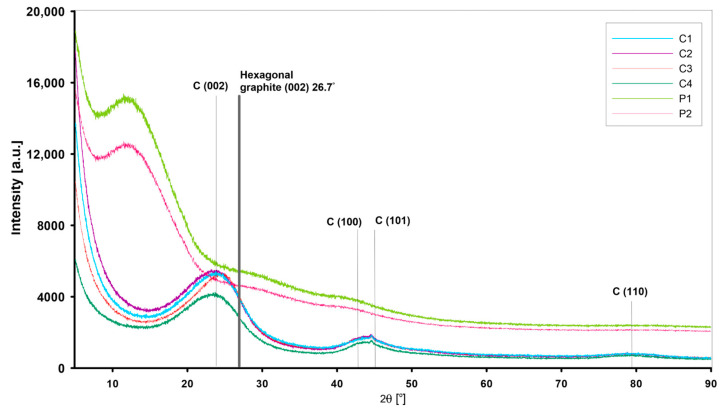
XRD patterns of mesoporous carbon (C1–C4) and polymeric materials (P1, P2).

In contrast to crystalline solids, which exhibit a regular and repeating pattern of atoms, the mesoporous carbons C1–C4 and the polymer materials P1, P2 show a lack of extensive ordering. Figure 6 shows the XRD patterns of the investigated mesoporous carbon materials (C1–C4). The first broad diffraction peak at 2θ = 23° can be attributed to the amorphous carbon structures of the graphite plane C(002). The diffraction peak at 2θ = 40–50° is due to the graphite structure and is labelled as C(100) and C(101). The broad nature of the XRD peaks indicates a low degree of graphitisation and identifies an amorphous carbon form. The position of the C(002) peaks for all carbon materials indicates a distance between the graphitic layers of 3.86 Å (greater than 3.35 Å for graphite (~26.7° of 2θ)). The amorphous structure of the polymer materials P1 and P2 was also confirmed. In this case, the XRD patterns (Figure 6) have no signals indicating impurities or other crystalline forms.

**Figure 7:**
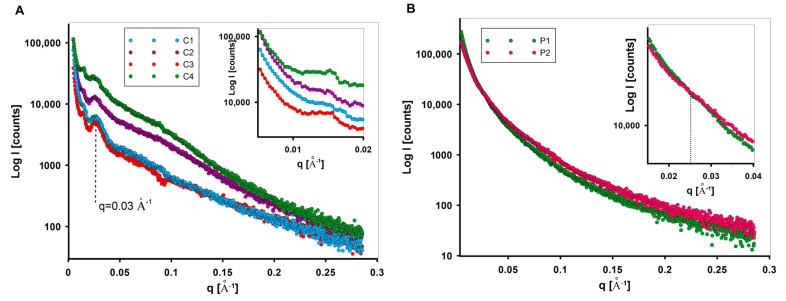
SAXS patterns of the investigated carbon C1–C4 (**A**), and polymer (**B**) materials.

The properties of nanostructured carbon and polymer materials are related to the structure and arrangement of the domains on the nanoscale. To understand the relationship between the size, shape, and arrangement of nanostructures and their macroscopic behaviour, SAXS analysis for all investigated materials was performed. Particles embedded in a matrix material must have an electron density unlike the matrix to become visible in SAXS. When neither pores nor particles are present in a dilute phase, it is impossible to assign the SAXS distribution unambiguously to either pores or particles. The visibility increases with the difference in the electron densities between the two materials. It is a consequence of equation [67].

(15) $$I_{1}\left(q\right)=I_{e}\left(q\right)4\pi {\left(∆\rho \right)}^{2}V\int_{0}^{d}\begin{array}{c} r^{2}\gamma_{0}\left(r\right)\frac{sinqr}{qr}dr \end{array}$$

where $I_{1}\left(q\right)$ is the intensity of small-angle scattering from a single particle corresponding to point scattering, $I_{e}\left(q\right)$ is scattering intensity by a single electron, ${\left(∆\rho \right)}^{2}\;$ is the difference between the electron densities of the particle and its surroundings, V is the volume of a particle, *d* is the maximum distance between points in a particle, (parameter referred to as the size of a particle), $\gamma_{0}\left(r\right)\;$ the correlation function for a single particle, and r the distance between two points in a particle. The above equation (Equation (15)) shows that the SAXS intensity is directly proportional to the square of the difference between the electron density of the particle and the surroundings. The SAXS effect therefore occurs regardless of whether the electron density of the particles is higher (particles) or lower (voids) than the electron density of the surroundings. This means that the sign of the contrast has no effect at all. Voids in a matrix material appear with the same intensity as the material particles in a void matrix. It is solely the judgment of the experimenter which parts of the sample are considered as “particles”. In the systems presented, the carbon material (or polymer materials) is considered as the matrix and the pores as scattering particles. The characterisation of such a system is based on other techniques (such as nitrogen adsorption/desorption at low temperature) that confirmed the presence of porosity in the nanometer range (suitable size of scattering objects for SAXS). The intensity of the scattering effect of the carbons C1–C4 (Figure 7A) differs in the initial phase of the SAXS curve. This may suggest various proportions of the scattering objects with mesoporous dimensions depending on the carbon type (in particular on the type of polymer matrix and the thermal treatment time during their synthesis), (inset in Figure 7A). This is supported by the porosity of the materials described by nitrogen adsorption/desorption data (Table 4). Here, sample C4 shows the highest share of porosity in the range of mesopores (pore sizes of 4.05 and 3.74 nm for D_BJH*ads*_ and D_BJH*des*_, respectively). The second highest SAXS intensity is demonstrated by the sample C2. Similarly, the pore sizes of 3.43 and 3.24 nm (Table 4) for D_BJH*ads*_ and D_BJH*des*_ distributions remain consistent with SAXS observations. Moreover, the SAXS curve for C4 runs at most up to the scattering vector value of 0.2 Å^−1^. When the observed feature of momentum transfer q is correlated with the length scale d probed in an X-ray scattering experiment by |q| = 2π/d, the size of inhomogeneity (d [Å]) can be calculated. Here, the position of q = 0.2 Å^−1^ corresponds to the d ~3.1 nm. From the above, sample C4 can contain objects with sizes above 3.1 nm in larger quantities than in other samples. Similarly, carbon C3 shows the fewest objects with such characteristics. Another feature of the tested porous carbons is the heterogeneity of the SAXS scattering curve. Some fluctuations in the curve may indicate characteristic structural elements. In all cases, the SAXS curves reveal the presence of inhomogeneities at position q = 0.025 Å^−1^. This corresponds to the objects of theoretical size ~25 nm. Taking into account the complex porous structure formed during carbon preparation (especially during the removal of the silica matrix when the extended porosity is formed), it is possible to obtain a hierarchical porous structure with elements of similar sizes. This can be confirmed by TEM images, which show extensive porosity with such characteristics [60]. Taking into account the course of the scattering curve, carbon materials differentiate into two groups. Taking into account the type of polymer matrix used for the synthesis of carbons (PE9400 for C1 and C3 and PE6400 for C2 and C4), they can determine the formation of porosity with different characteristics. Differences in the porous structure translate directly into the nature of the interaction of the nanostructure with X-ray radiation at low angles and determine the shape and course of the experimental curve (Figure 7A). In the case of carbon materials, these differences are quite significant.

By analysing the shape and extent of X-ray scattering in Figure 7, it can be found that the scattering effect of the carbon (Figure 7A) and polymer materials (Figure 7B) is quite different.

Polymer materials generate a significant SAXS effect in the range of small diffraction angles, which suggests the presence of nano-objects involved in this phenomenon. They exhibit a polydispersive nature that highlights the absence of any structural ordering or enhanced heterogeneity features. The scattering intensities are significant, although the shape of the experimental curves is very similar. A characteristic feature of the polymer samples (Figure 7B) is the intersection of the SAXS curves at the position q = 0.025 Å^−1^. This could indicate the differentiation of the proportion of objects with sizes of 25 nm. In the initial region of the SAXS curve, i.e., below the value q = 0.025 Å^−1^, the scattering intensity for the sample P1 is higher, while after passing this point the intensity of the SAXS curve for the sample P2 is slightly higher. This indicates a greater proportion of larger pores (up to 25 nm) for the sample P1 and a greater proportion of smaller structures for the sample P2.

**Figure 8:**
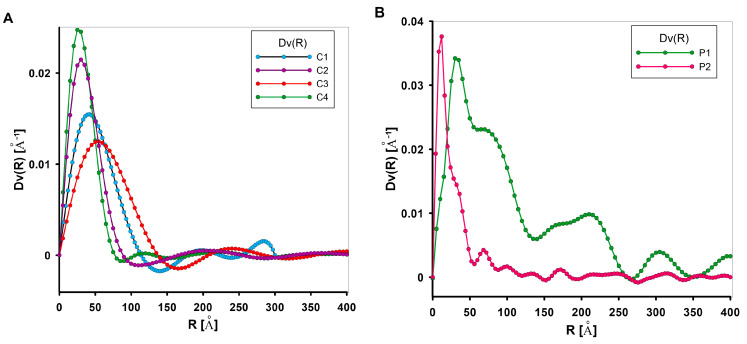
Comparison of volume size distribution function (Dv(R)) of the scattering heterogeneities in investigated materials: the mesoporous carbons C1–C4 (**A**), and the mesoporous polymer (**B**) materials.

From the small-angle scattering data, the size distribution of the scattering objects (pores) Dv(R) can be determined if they have approximately the same shape. It should be emphasised that changing the shape of scattering objects has a smaller impact on the shape of the small-angle scattering curve than changing their size. This size can be determined by the length of the dimension characteristic of a given shape. For spherical objects, the characteristic dimension may be their radius or diameter. If R is the value of the characteristic dimension of the scattering particle, and D(R) is the function of the particle size distribution, then D(R)dR will represent the probability of occurrence of objects with dimensions in the range R to R + dR. Figure 8 shows the scattering objects size distribution function, where DV(R)dR is proportional to the volume of pores ranging in size from R to R + dR. The Dv(R) curves presented in Figure 8 determine the volume-weighted particle (or pore) size distribution Dv(R) from the scattering curve of an ensemble of spherical objects (or pores) having a homogeneous inner electron density distribution, and not showing interparticle interaction effects. The Dv(R) function refers to the volume-weighted particle size distribution (‘volume distribution’). It may be seen as a histogram of the radii R of the particles (or pores) that are present in the sample. The height of the Dv(R) function is proportional to the volume (not to the number) of particles that can be found within a given size interval. In the case of carbon samples, Dv(R) curves of carbons (Figure 8A) have a single, well-pronounced peak, which enables one to determine the most probable particle size (by volume) and stable solutions from the size distribution analysis. In the case of polymer samples (Figure 8B), the given sphericity criteria may not be accurately defined and deviate from Gaussian or Log-normal functions. In this case, the well-pronounced peak is disturbed, as evidenced by fluctuations in the curve in the area above 50 Å. Assuming that the tested materials are a porous system, it should be taken into account that all pores present are determined, including closed pore systems that are normally not accessible to the analysing molecules, such as nitrogen molecules. This results in possible differences in their size distributions as well as in the determined S_SAXS_ interface.

The interphase surface S_SAXS_ refers to the boundary between two materials or phases in a sample. This surface can be characterized by analysing the scattering intensity of X-rays as they pass through the sample. Porod’s law describes the asymptotic behaviour of a scattering curve towards high scattering angles. Scattering curves of particle systems and porous materials with smooth surfaces decay proportionally to q^–4^ at large angles: I(q) ≈ K_4_/q^4^ for non-smeared data (K_4_ is the Porod constant). For slit approximation, the decay is proportional to q^–3^ due to the slit-smearing effects (I(q) ≈ K_3_/q^3^ for smeared data where K_3_ is the smeared Porod constant). Moreover, the surface-to-volume ratio is based on the Porod constant K and the scattering invariant Q as S/V = (K_3_/Q)·4. The invariant Q was estimated using linear extrapolation to q = 0 as a function I·q^n^ vs. q where n = 1 for smeared data. The Q values were estimated automatically during SAXS data treatment (Porod analysis) by EasySAXS (PANalytical) software.

**Figure 9:**
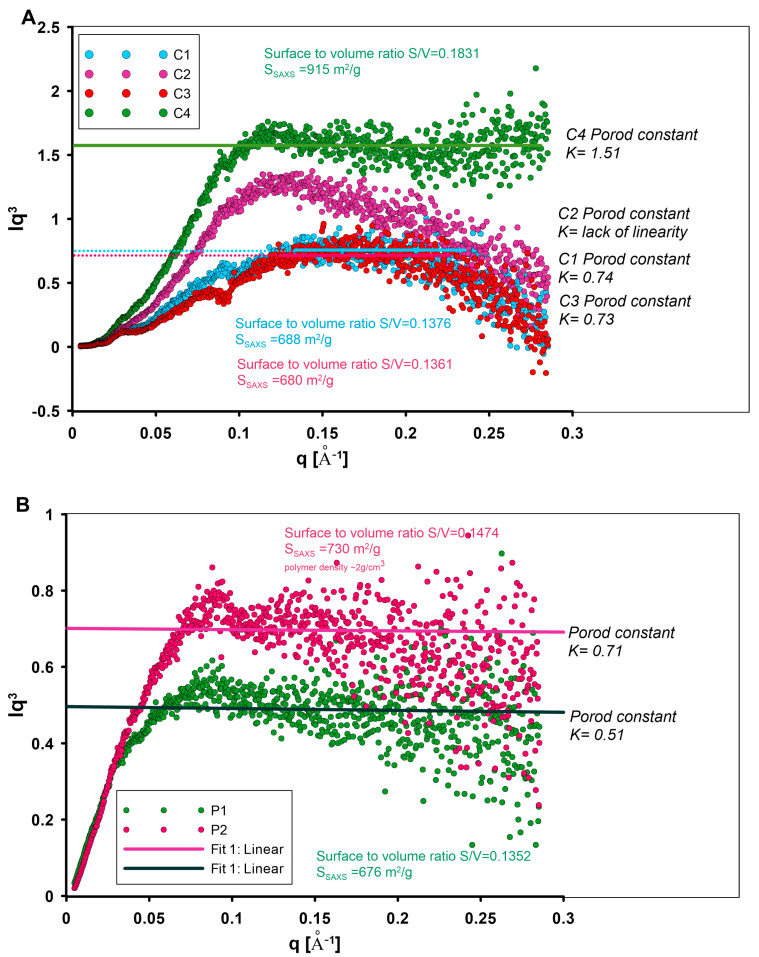
Porod plots were calculated for investigated porous carbons (**A**) and polymer materials (**B**). The asymptotic range of the Porod curve was approximated by a linear function for the Porod constant. Specific surface S/V was calculated from I(0), Porod invariant, and asymptotics. The S/V values were used for the calculation of S_SAXS_ from the equation: S_SAXS_ = (10,000×$\frac{S}{V})/d$ where d is the bulk density of the materials.

The Porod constant was determined to ensure the asymptotic intensity decay proportional to q^−3^ at higher angles according to the Porod law for homogeneous particles. A high degree of linearity in the Porod range may indicate a well-developed interphase boundary without the current phenomenon of blurring. The Porod plots calculated for all tested samples can be found in Figure 9. Sample C4 shows the lowest deviation from the linearity law among the tested carbon materials. The linearity range of the function I·q^3^ = f(q) for the sample C4 covers q values from 0.125 Å^−1^ to almost the end of the curve. For this sample, the very last part of the curve even rises slightly upwards. Although such a positive deviation is small, it may indicate the presence of microporous regions within the interface that are responsible for additional scattering. The samples C1 and C3 show a very narrow linearity range (q from 0.13 to 0.23 Å^−1^). For these samples, this linearity range is subject to a significant negative deviation from the linearity assumptions. It is visible as a reduction in scattering, particularly at high angles. Therefore, the values for the specific surface area may be distorted. Such a situation assumes that a quasi-two-phase system has a diffuse phase boundary or a transition zone (i.e., a boundary layer) of average thickness between two general phases. In the case of sample C2, the interface layers are expected to be highly blurred. In this case, the negative deviation from the linear curve is so large that the Porod constant cannot be determined with a reasonable value. The polymer samples P1 and P2 illustrate the scattering by a two-phase system with a sharp boundary between the electron densities in each phase. Comparing the Porod curves for polymers and carbon materials, the latter shows a significantly higher phase contrast. 

The specific surface areas calculated from the Porod analysis agree well with the BET results (taking into account the general reciprocal relationship of the size of the interface). However, the SAXS values are slightly higher than the BET values. A possible reason for this is probably that SAXS can also detect closed pores and the nanostructured surface of the particles, which are not accessible to the BET method. For porous materials, SAXS therefore provides access to the closed porosity. The largest difference in area value obtained with these two methods concerns sample C4 and corresponds to ~260 m^2^/g, indicating a rather significant closed porosity that may not be involved in the adsorption process. For the other samples, the closed porosity value is similar and amounts to approximately ~150 m^2^/g–180 m^2^/g.

#### 3.1.3. Surface Morphology of Adsorbents

**Figure 10:**
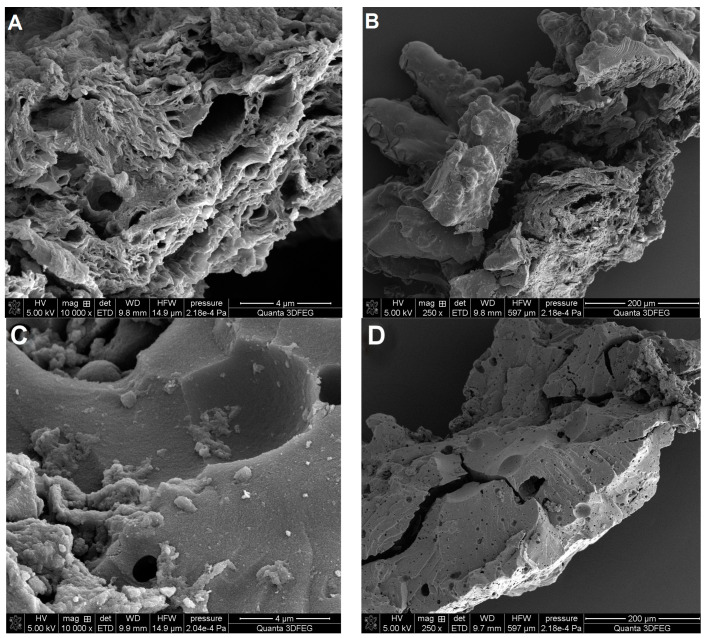
SEM images for the carbon materials C3 (**A**,**B**) and C4 (**C**,**D**) at two selected magnifications.

**Figure 11:**
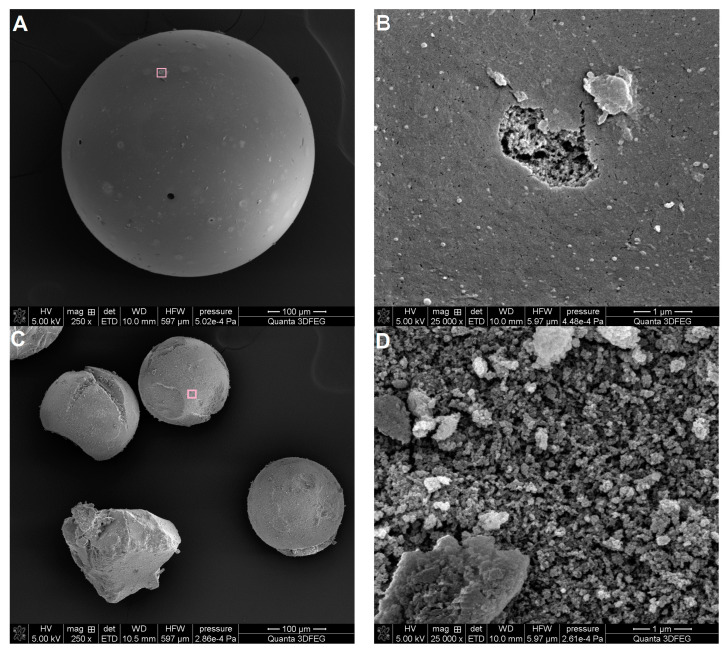
SEM images for the porous polymer P1 (**A**,**B**) and P2 (**C**,**D**) at two selected magnifications. SEM images (**B**,**D**) are an enlargement of the area marked with a square.

Scanning electron microscopy was used to image the surface morphology of investigated carbon and polymer materials (Figure 10 and Figure 11). The morphology of both carbons C3 and C4 is differentiated (Figure 10). The surface morphology of the carbon C3 is characterised by uneven cavities and fine open pores including layered systems and crevices. In the case of the carbon C4, symmetrical forms of porous structures are visible, and the entire carbon material is much more compact and tight. The morphological images are in close correlation with the porosity analysis using low-temperature nitrogen adsorption–desorption and SAXS results. The SEM analysis combines the conclusions regarding the larger pore sizes for sample C3 (Figure 5B and Figure 8A), the amount of closed porosity (the largest for sample C4), and the size of the interfacial surface (also the largest and with the sharpest edge for the sample C4), which is shown in Figure 9.

Morphologically, the polymer materials P1 and P2 constitute spheres with dimensions of ~500 μm and ~300 μm. The difference concerns the quality of the spheres for the sample P2 and the quality of their formation. The polymer material P1 consists of spheres with a uniform and smooth surface, under which, as shown in Figure 11B, there is a porous structure. The polymer P1 consists of spheres with a greater number of deformations and a disturbed spherical structure. Similar to the sample P1, the interior of the sphere is also made up of a porous polymer structure (Figure 11D).

#### 3.1.4. Surface Chemistry of Adsorbents

In addition to structural features such as the surface area of the adsorbent, and porosity properties, which determine the effectiveness of the process, and the transport of adsorbates, the chemistry of the surface and its affinity for potential adsorbates are specific factors in the adsorption process. In this work, X-ray photoelectron spectroscopy (XPS) was used as a quantitative technique to measure the elemental composition of the surface and to determine the bonding states of the elements.

**Figure 12:**
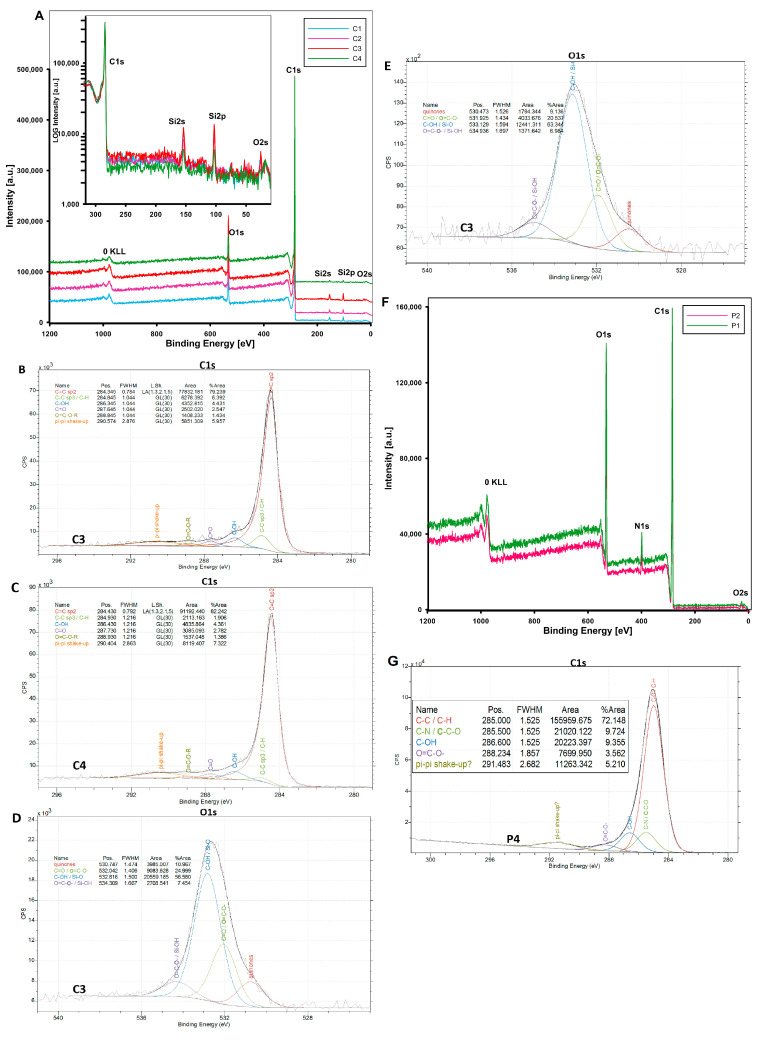
(**A**) Survey scan XPS spectra of the samples C1–C4. In the main figure, the XPS spectra have been moved apart on the ordinate scale for better visibility. In the inset, the 0–300 eV region has been enlarged and the spectra are in the original intensity scale. (**B**) High-resolution core-level spectra from the C1s region for sample C3, and (**C**) high-resolution core-level spectra from the C1s region for sample C4 with the corresponding deconvolution components for the investigated samples. The model of the C1s region for the samples C3 and C4 was based on Biesinger’s work [68]. (**D**) High-resolution core-level spectra from the O1s region for sample C3, and (**E**) high-resolution core-level spectra from the O1s region for C4. (**F**) Survey scan XPS spectra of the samples P1 and P2, and (**G**) a high resolution of C1s signals.

Figure 12 shows the survey scan and high-resolution spectra of mesoporous carbon surfaces (C1–C4). The details of the XPS analysis of investigated samples are presented also in Table 5.

The XPS peaks of the main elements detected on the very top surface (1–10 nm) were labelled on the XPS spectra. In the case of mesoporous carbon materials, the O1s, O2s, C1s, Si2s, and Si2p signals were identified for all materials. Thus, the composition of the tested materials was confirmed, while a silica phase content of several percent was observed as a residue from the synthesis of carbon materials. The content of the silica phase is not high, and the intensity of the 2s and 2p peaks (the highest for sample C3) is reflected in the percentage shown in Table 5 (maximum value 4.4% for sample C3). For the adsorption process of derivatives of thiazine and triarylmethane, the most important issue is the surface chemistry and functional groups related to carbon atoms that can effectively participate in such a process. For this purpose, high-resolution XPS analysis was performed. Because this work focuses on a detailed description of the adsorption of dyes on the carbons C3 and C4, the detailed XPS analysis will also be limited here for these two materials. Two selected carbons (although the remaining carbons C1 and C3 have similar results) show similar spectrum components, and the difference mainly concerns their mutual content. The main peaks of C1s at 284.3 and 284.8 eV are attributed to the sp2 carbon of C=C bonding and the sp3 carbon of C–C in the graphitic structure. The proportional content of these forms is 79.3% and 82.2% (C=C), and 6.4% and 1.9% (C–C) for the carbons C3 and C4, respectively. Moreover, the C1s spectrum includes three peaks attributed to the oxygen-containing functional groups. Peaks at 286.3 eV, 287.6 eV, and 288.5 are attributed to the carbon atoms in the C–OH/C–O–C, C=O, and O=C–O–R functional groups, respectively. Despite the visible difference in the O1s content for the analysed coal samples (Table 5), where for sample C3 the %At of O1s was 13.2% and for sample C4 it was 7.3%, the atomic analysis of the C1s carbon and the oxygen groups associated with it (Figure 12B,C) turned out to be very close. Due to the important role played by oxygen groups in the dye adsorption process, the energy states of the O1s atom were analysed (Figure 12D,E). In this case, the sample C3 contains (as %At) a larger share of individual oxygen groups compared to the sample C4. The characteristics of the individual components of the O1s signal after its deconvolution indicate the presence of quinones (~530 eV), C=O or O=C-O- groups (~532 eV), C-OH and Si-O groups (~533 eV), as well as O=C-O-/Si-OH groups (~534–535 eV) for all carbon samples. All identified groups are acidic and fully reflect the strongly acidic nature of the adsorbent, which was experimentally determined using potentiometric titration. This confirms the adsorption potential towards cationic adsorbates (selected dyes).

XPS analysis of polymer materials revealed the presence of oxygen (O1s), nitrogen (N1s), and carbon (C1s) as primary elements on the surface of the polymer materials. The total atomic content of each element is consistent with the expected structure resulting from the combination of monomers shown in Figure 2 (e.g., the highest oxygen content for the sample P1 (19.6 %At) obtained from oxygen-rich monomers (VP and TRIM) and a higher carbon content (% At) for the sample P2 (92.3 %At) obtained from carbon-rich monomers (VP and DVB)). The confirmed presence of π-π- forms can determine the basic nature of the polymeric materials.

**Figure 13:**
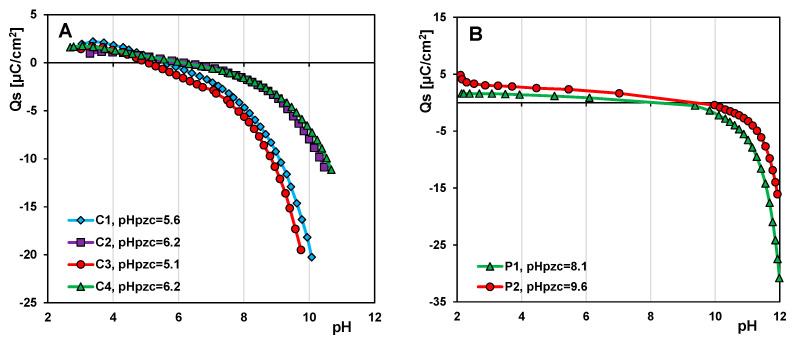
Dependences of surface charge density on pH for carbons (**A**) and polymer materials (**B**).

To evaluate the acid/base properties of the carbon and polymer materials, potentiometric titration experiments were performed. Figure 13 shows the dependence of the surface charge density on the pH value of the solution for the adsorbents investigated. The values obtained for the zero point of the charge pHpzc are in the range of 5.1–6.2 for carbon samples. It can be seen that the surface of the carbons was negatively charged under the test conditions (neutral pH solutions). Of all the carbons, C1 and C3 had the strongest charge, which is due to a larger number of acidic moieties on their surface. The data analysis for the polymers shows their basic character with pH_PZC_ values of 8.1 and 9.6 for P1 and P2, respectively. The higher pH_PZC_ value for polymer 2 is related to the strong basicity of the DVB monomers, which are absent in P1.

### 3.2. The Adsorption Properties of the Mesoporous Materials

**Figure 14:**
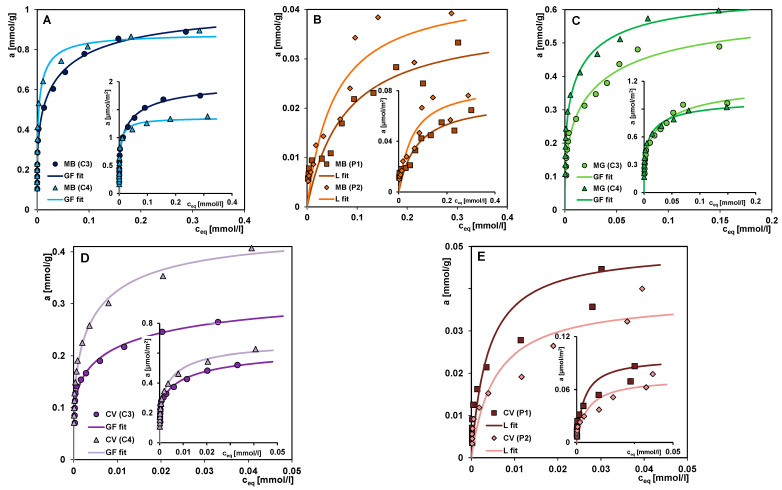
Comparison of the adsorption isotherms for (**A**) methylene blue on C3 and C4, (**B**) methylene blue on P1 and P2, (**C**) malachite green on C3 and C4, (**D**) crystal violet on C3 and C4, and (**E**) crystal violet on P1 and P2. The lines correspond to the fitted Generalized Langmuir equation (Generalized Freundlich (GF) and Langmuir (L) equations). The inset figures show the adsorption isotherms in the reduced form *a/S_BET_* vs. *c_eq_*.

The adsorption properties towards cationic dyes, which are derivatives of thiazine (methylene blue; MB) and triarylmethane (crystal violet; CV), were investigated for selected materials: the mesoporous carbons C3 and C4 as well as the mesoporous polymers P1 and P2. For carbon materials, additional adsorption studies with another derivative of triarylmethane (malachite green, MG) were carried out. Figure 14A–E show the adsorption isotherms of the individual dyes on carbon and polymer materials in the standard form *a* vs. *c_eq_* and the reduced form *a/S_BET_* vs. *c_eq_*. Analysis of Figure 14A,B suggests that methylene blue is comparably adsorbed by the carbon C3 which is a material of mixed porosity (microporous and mesoporous) and the mesoporous carbon C4, in comparison to the mesoporous polymeric materials P2 and P1 for which the weakest adsorption is found. The values of a maximum adsorption capacity (a_m_) determined based on the Generalized Langmuir equation (GL) for these materials equal to 1.01; 0.88; 0.044 and 0.038 mmol/g, respectively. Such a significant difference in the adsorption of methylene blue on different types of materials (carbon or polymer) is the result of several closely related factors, i.e., (i) affinity of the solid for the dye under given experimental conditions; (ii) surface charge of the adsorbent; (iii) chemical form of the adsorbate molecule; (iv) porosity of the adsorbent; and (v) molecular dimensions of the adsorbate and its three-dimensional structure.

The high affinity of carbons for methylene blue is frequently observed [60,69,70,71] and can be attributed to (i) strong attractive interactions between the deprotonated functional groups on the adsorbent surface and the ionized adsorbate; (ii) π–π interactions of the adsorbent graphene layers with the adsorbate aromatic polyring; (iii) free dispersion interactions (London forces); (iv) hydrogen bonds between nitrogen, sulphur, or hydrogen atoms (methyl groups) of the adsorbate molecule and hydrogen or oxygen atoms belonging to the adsorbent surface groups. In the case of polymers, their much lower affinity for methylene blue is due to (i) repulsive electrostatic forces between the weakly positively charged adsorbent particles and the adsorbate cation; (ii) lack of or slight interactions resulting from differences in the distribution of electron density in aromatic systems (X-ray diffraction studies). Due to the chemical structure of polymers and methylene blue, free dispersion interactions and hydrogen bonds are highly probable. The influence of the surface charge of polymers on the affinity towards ionisable dyes has been discussed in many works [72,73,74].

The slope of the adsorption isotherm in the range of low equilibrium concentrations is a measure of adsorbent–adsorbate affinity. This adsorption property is also expressed by the value of the equilibrium constant (log K) obtained by fitting the theoretical curve to experimental data. For the adsorption systems discussed, this parameter is in the range of 1.12–1.62 which indicates high affinity adsorbents towards adsorbate. However, it should be noted that log K is significantly overestimated for systems with polymers due to the type of theoretical isotherm chosen (Langmuir isotherm). In turn, the results of fitting using the Generalized Freundlich (GL) equation for dye-polymer systems give more realistic values of this parameter, but the values of maximum adsorption capacity (a_m_) are overestimated.

By analysing the structural (from nitrogen adsorption/desorption and SAXS measurements) and surface chemical properties (from potentiometric titration and XPS measurements) of the two groups of materials, it can be concluded that their significantly differentiated adsorption efficiencies towards methylene blue are mainly due to a difference in surface charge. Under the experimental conditions, methylene blue was in the ionized form with a positive charge, while the activated carbons were characterized by a negative surface charge due to partial ionisation of the acidic groups. The different charges of the contacting species promoted adsorption by electrostatic interactions. It can be assumed that the presence of silica in the carbon materials as a residue of the incompletely removed matrix did not affect dye adsorption (a weak mutual affinity). In the case of the polymeric materials characterized by a weakly positive charged surface, contact with the adsorbate cations resulted in repulsive electrostatic interactions which also limited other possible adsorption mechanisms.

Regarding the influence of the solid porosity on the methylene blue adsorption, a correlation between the adsorption capacity and the pore size is observed for both groups of materials. Methylene blue, as a relatively small molecule with a linear structure (Figure S1 in Supplementary Materials), is adsorbed more strongly in smaller pores (micropores and small mesopores) than in larger pores (medium and large mesopores). Therefore, carbon C3 turned out to be a relatively more effective adsorbent (a_m_ = 1.01 mmol/g) than carbon C4 (a_m_ = 0.88 mmol/g), despite its smaller specific surface area and total pore volume. These parameters for C3 and C4 were 508 m^2^/g and 0.29 cm^3^/g and 649 m^2^/g and 0.65 cm^3^/h, respectively. In the group of polymers, P2 (a_m_ = 0.044 mmol/g) was more effective than P1 (a_m_ = 0.038 mmol/g). The V_t_ values for P2 and P1 are 0.75 cm^3^/g and 1.06 cm^3^/g, respectively (the specific surface area of both polymers is the same). It can be concluded that when methylene blue is adsorbed in porous solids, which are characterized by different mesopore sizes, part of the pore space remains unfilled with the adsorbate. This is well reflected in the isotherms course when plotted in reduced form *a/S_BET_* vs. *c_eq_* (inset figures in Figure 14).

Figure 14C presents the adsorption isotherms of malachite green on carbon materials. Malachite green molecules are larger and have a more developed spatial orientation than methylene blue ones (Figure S1 in Supplementary Materials), so a sieving effect in the system with carbon C3 can be assumed. The sieving effect enables the diffusion of the dye into the pore space of the adsorbent with sizes smaller than the adsorbate or with narrow entrances. The adsorption capacity values of the carbons C3 and C4 towards malachite green are 0.60 and 0.65 mmol/g, respectively, indicating a reverse trend in the efficiency of the adsorbents compared to their behaviour for methylene blue. Lower log K values (0.96 and 1.19) indicate again a decrease in the affinity of the activated carbons when the adsorbate changes from a thiazine derivative to a triarylmethane derivative. The changes in affinity between adsorbent and adsorbate may be related to the difference in the chemical structure of two dyes, i.e., the presence of a triphenylmethyl group, which is a major steric hindrance in the malachite green molecule, and a phenothiazine scaffold with a sulphur and nitrogen atom in the methylene blue molecule. The difference also concerns the number of double bonds and the distribution of the positive charge in the molecule, which originates from the secondary amino group. It can be assumed that the relative proportion of the individual intermolecular interactions involved in the adsorption mechanism of the two dyes differs significantly.

By comparing the adsorption isotherms of crystal violet on carbon and polymer materials (Figure 14D,E), one can conclude that polymers are worse adsorbents for crystal violet, similar as it was in the case of methylene blue, but the difference in adsorption efficiency is not as significant. Crystal violet is an analogue of malachite green, which contains an additional second-order amino group on the phenyl ring in its structure, which leads to an enlargement of the molecule (Figure S1 in Supplementary Materials). In the group of carbons, a deeper differentiation of their adsorption capacities is observed as a result of the sieving effect, which excludes large adsorbate molecules from the small pores of the adsorbent C3. The adsorption capacity values of the carbons C3 and C4 for crystal violet are 0.33 and 0.44 mmol/g, respectively. In the group of polymers, P1 reveals a higher adsorption efficiency for the dye. The values a_m_ and log K are for P1: 0.050 mmol/g and 2.41 and for P2: 0.038 mmol/g and 2.26. It is worth noting that the trend is reversed compared to systems with methylene blue. The main reason for this is the change in the relations between the adsorbate size and the pore diameters of the polymer P1. Moreover, the higher polarity of the polymer P1 (both monomers, i.e., 1-vinyl-2-pyrrolidone and trimethylpropane trimethacrylate are polar) determines its higher affinity for crystal violet compared to the polymer P2 with lower polarity (polar or non-polar monomer: 1-vinyl-2-pyrrolidone and divinylbenzene, respectively).

**Figure 15:**
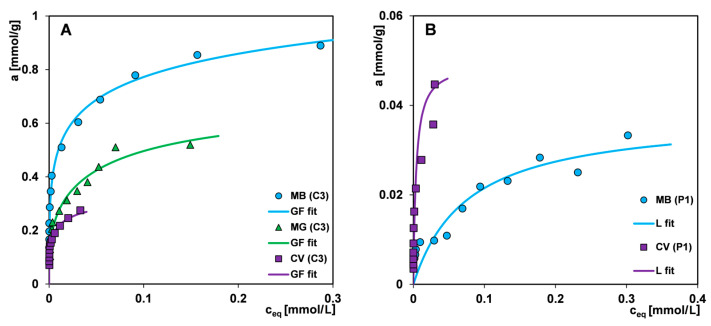
Comparison of the adsorption isotherms for various dyes on selected activated carbons (**A**) and polymer material (**B**).

Figure 15A,B compare the adsorption isotherms of three dyes on selected activated carbon and polymer materials. In general, different trends can be observed in the affinity of the two material groups for dyes. The efficiency adsorption of the carbon decreases in the following order: MB > MG > CV, and of the polymer: CV > MB. The observed decrease in the amount of adsorbed dyes on the carbon material is determined by the increase in the size of the subsequent adsorbates and their different spatial shape. The porous structure of the mixed type (micro- and mesopores) carbon C3 gives the highest efficiency in the removal of pollutants characterized by a small molecular size, such as methylene blue. The effect is enhanced by the linear structure of the adsorbate, which results in larger packing in the pore spaces. For adsorbates with larger molecule sizes and non-linear structures, such as malachite green and crystal violet, some pores are excluded from participating in the adsorption process due to the sieving effect. The greater adsorption of a larger molecule with a more extended spatial structure, i.e., crystal violet on the surface of the polymer material, seems to be due to the greater compatibility of the adsorbate with the pore size (Table 3 and Figure S1 in Supplementary Materials). Moreover, it should be added that the course of the isotherm CV (P1) in Figure 15B may indicate an incorrect selection of the concentration range for CV dye in combination with the used sorbent dosage. The same isotherm is compared in Figure 14E with a CV adsorption isotherm on polymer P2 (the range of the X-axis adjusted to the values of experimental data) where we can observe the equilibrium establishing.

To describe the experimental adsorption isotherms the Generalized Langmuir (GL) equation was applied. Generally, the adsorption of dyes on carbon and polymeric materials may be described by special cases of GL, i.e., the General Freundlich (GF) and Langmuir (L) equations, respectively (Table 6). Low values of parameter m for adsorption systems with carbons (m~0.22–0.32) means a high heterogeneity effect. For the remaining systems (with polymeric materials), the values of parameters m = 1 and n = 1 suggest that these adsorbents are characterized by energetic homogeneity.

To obtain comprehensive adsorption studies of methylene blue, malachite green, and crystal violet on carbon and polymer materials, the scope of the experimental work was supplemented by kinetic measurements. It is widely known that the applicability of porous solids in water and wastewater treatment technologies depends on both their adsorption capacity and the process rate. Sometimes, slow adsorption kinetics is a critical factor that makes a particular adsorbent unusable in real applications.

Due to the significant difference in the adsorption capacities of both groups of adsorbents, the same experimental parameters were used for the kinetic studies of the dye-activated carbon systems and the dye-polymer systems, such as adsorbate concentration, solution volume, mixing rate, and temperature, while the adsorbent mass was treated as a variable parameter. For adsorption systems with polymers, a solid weight was exactly 7.5 times larger than this carbon (m~0.15 g and 0.02 g, respectively).

**Figure 16:**
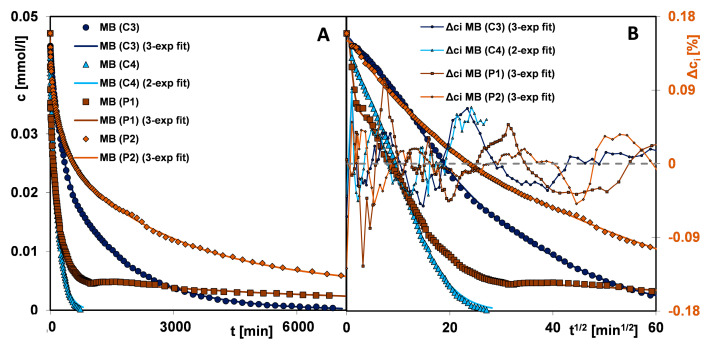

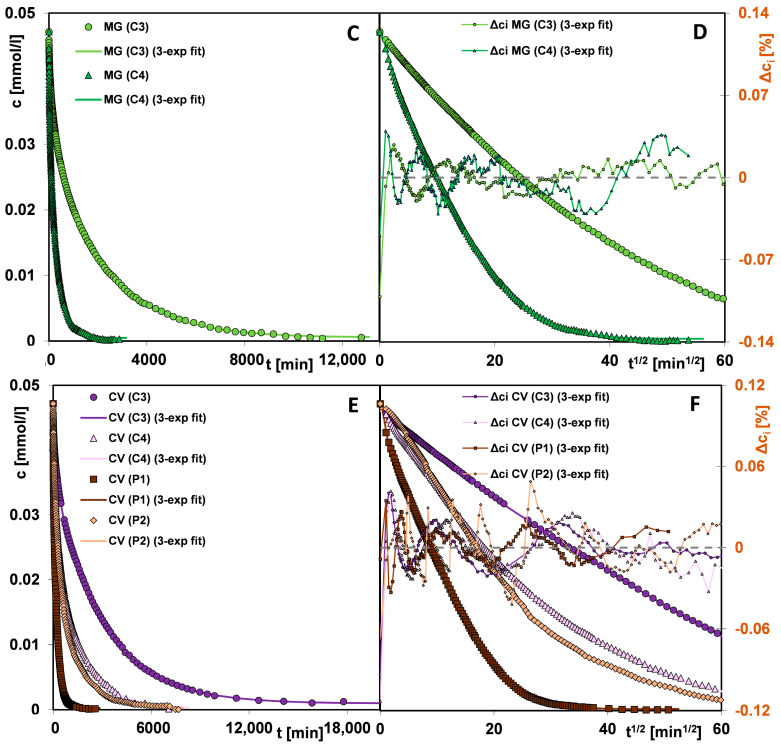
Comparison of adsorption kinetics for (**A**,**B**) methylene blue, (**C**,**D**) malachite green, and (**E**,**F**) crystal violet on selected activated carbons and polymer materials at concentration—time and concentration—square root of time coordinates. The lines correspond to the fitted m-exponential equation.

Figure 16A–F show the kinetic curves of a given dye adsorption on the materials C3, C4, P1, and P2 in the form of adsorbate concentration as a function of time and adsorbate concentration as a function of the square root of time. The use of the c-t_1/2_ scale allows for more precise observation of the initial course of the kinetic curve. It is well known that the adsorption process in a liquid–solid system comprises several stages: (i) mass transfer of adsorbate molecules by diffusion from a bulk solution to the external surface of a solid, (ii) intramolecular diffusion (diffusion in adsorbent pores into its internal surface), (iii) adsorption according to physical or chemical mechanisms at the active sites of the solid surface [75]. The slowest stage defines the course of the entire process. Therefore, the kinetics of dye adsorption is closely correlated with the structural properties of the adsorbents and the molecular size of adsorbates. In the group of carbons, the adsorption process on the carbon C4 is the fastest. This is typical for mesoporous materials, which are additionally characterized by a high affinity for the adsorbate. One can observe the extended linearity of the concentration profiles as a function of the square root of time, which proves the high dynamics of dye loss in the bulk solution caused by its efficient diffusion into the pore space and the occupation of adsorption sites on the solid surface. In the case of the mixed type (micro- and mesoporous) carbon C3, the stage of intramolecular diffusion of the adsorbate is a critical one that slows down the adsorption process. With the increase in the adsorbate molecule size and the development of its shape, the linearity region of kinetic profiles is shortened. For example, the adsorption of methylene blue, a molecule that is characterized by the smallest size and linear shape, gives a profile with the largest range of linearity, while the crystal violet adsorption with the largest molecular size and spatially extended shape is significantly shortened. Based on these observations, one can indicate diffusion from a bulk solution and intramolecular diffusion as critical stages for the kinetics of malachite green and crystal violet adsorption. The differentiated adsorption rate on the mesoporous carbon C4 and the mixed type (micro- and mesoporous) carbon C3 is revealed by the values of the half-time t_1/2_ determined in the optimisation procedure using the multi-exponential equation (this equation among all ones turned out to best describe the adsorption kinetics of tested systems; the results are collected in Table S1 in Supplementary Materials). The t_1/2_ parameter is the time needed to achieve a 50% change in solution concentration and for the systems with C4 and C3, it is 99 and 416 min, respectively (the methylene blue adsorption); 116 and 710 min, respectively (the malachite green adsorption); and 406 and 1311 min, respectively (the crystal violet adsorption). One can also notice a huge difference in the times needed to reach the equilibrium state, which is related to the porosity characteristics of the adsorbents. The carbon C4 with wider pores enables it to reach equilibrium 9.4 times (the methylene blue adsorption), 4.5 times (the malachite green adsorption), and 2.5 times (the crystal violet adsorption) faster compared to the carbon C3.

The adsorption kinetics of dyes in the polymer group show a similar trend as in the carbon group. The polymer P1 with larger pore sizes enables a faster process of pollutant removing from an aqueous solution than the polymer P2. The half-time t_1/2_ values in the systems with P1 and P2 are 87 and 570 min, respectively (the methylene blue adsorption); and 92 and 337 min, respectively (the crystal violet adsorption). During the methylene blue adsorption on P1, a state close to equilibrium is reached after approx. 4500 min, while in the case of P2, the experiment time is insufficient to determine its value. For the crystal violet—P1 system and the crystal violet—P2 system adsorption remains at the same level after approx. 2100 and 7100 min, respectively, which corresponds to a difference of 3.4 times.

By analysing the concentration profiles for two groups of materials, a correlation between the dye adsorption rate, the affinity of the adsorbent for the dye, and the adsorbent porosity can be seen. The use of a polymer weight 7.5 times higher than that of activated carbon compensates for a weak affinity of the adsorbent for methylene blue to such an extent that the kinetic profiles on the polymer P1 and the carbon C4 have very similar course. However, some of the adsorbate remains in the solution in an unbound form. The situation is slightly different during the crystal violet adsorption towards which the polymer has a higher affinity. Even though the crystal violet molecule is larger than the methylene blue molecule and is additionally spatially expanded, the adsorption rate is higher on the polymer P1 than on the carbon C4. For both systems, the equilibrium uptake u_eq_ equals unity. One can see the similarity in the kinetics of crystal violet on the carbon C4 and the polymer P2, with a smaller share of larger pores than the polymer P1.

**Figure 17:**
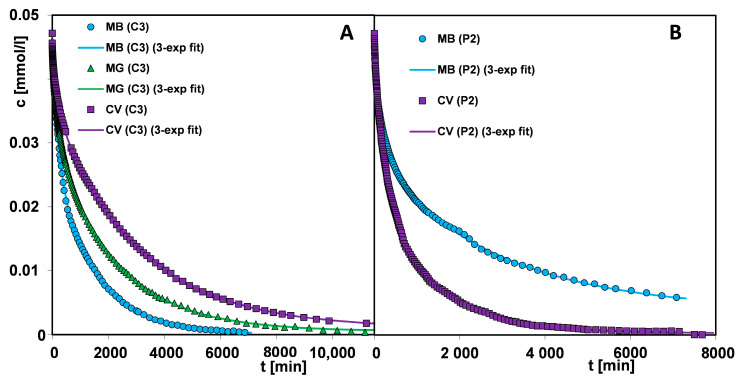
Comparison adsorption kinetics for various dyes on selected activated carbon (**A**) and polymer material (**B**).

The observations described above are confirmed by the kinetic profiles of dyes adsorption on the selected carbon and the polymer materials (Figure 17A,B). There is a clear change in the trend of the dye adsorption rate when changing adsorbent, i.e., for the carbon C3 there is an increase in the adsorption rate consistent with the decrease in the adsorbate size and shape, and for P2 there is an opposite situation, due to the difference in adsorbent–adsorbate affinity.

**Figure 18:**
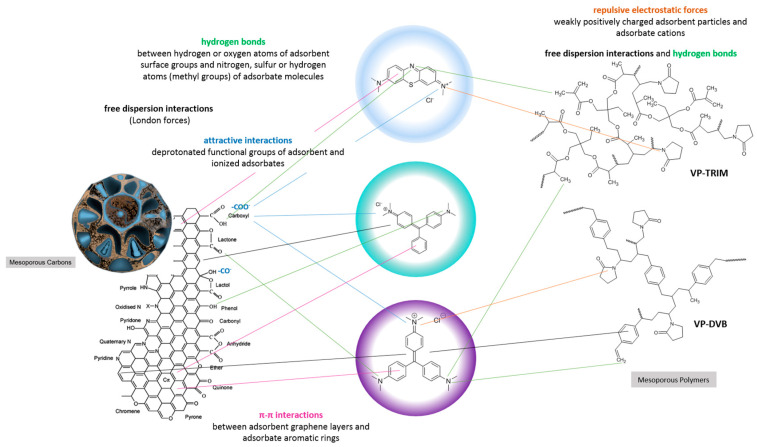
Schematic diagram of adsorption mechanism.

The adsorption kinetics of tested systems was analysed by applying the classical equations e.g., first-order (FOE), second-order (SOE), and less popular equations, e.g., mixed 1,2-order (MOE), multi-exponential (m-exp), fractal-like MOE (f-MOE). The values of the parameters of various kinetic equations are collected in Table S1 in the Supplementary Materials. One can observe a good agreement in the values of log k, t_0.5_, and u_eq_ defined using the multi-exponential equation and the fractal-like MOE equation. For these equations, a much better quality of fitting to the experimental data than for the other kinetic models was also revealed (Figure S2 in Supplementary Materials). It is confirmed by the values of relative deviations (SD (c/c_0_)) and convergence coefficient (1-R^2^). Significant deviations of FOE/SOE/MOE, but much smaller for m-exp. and f-MOE, suggest non-ideality of adsorption systems. This is closely related to the concept of kinetic system fractality (f-MOE) and the assumptions of the semi-empirical multi-exponential equation about the energetic or structural heterogeneity of adsorption systems. The diagram of the adsorption mechanism of the discussed systems is shown in Figure 18.

## 4. Conclusions

- The proposed synthesis methods allow mesoporous carbon and polymer materials with differentiated characteristics to be obtained. Differences in the properties of the porous structure were determined by nitrogen adsorption and SAXS measurements. These differences concerned the specific surface area, the presence of micro-sized pores, the content of meso-sized pores, and levels of closed porosity, influencing adsorption capacity. The tested materials show activity in the interaction with X-ray radiation in the range of small diffraction angles. The SAXS effect allowed various proportions of the scattering objects to be determined, as well as their size distribution and the quality of the carbon/pores or polymer/pores interfacial area. The phenomenon of blurring of the interfacial area was confirmed in the case of some samples (e.g., sample C2) or their sharp boundary (as in the case of polymer samples). The content of closed porosity was determined.
- The surface properties of mesoporous carbon and polymer materials were determined by identifying the surface functional groups and their atomic content (XPS method), and surface charge (potentiometric titration method). The acidic nature of the surface of carbon materials was confirmed due to the presence of functional groups such as C=O or O=C-O-, C-OH, Si-O-, O=C-O-, and Si-OH groups. These groups give the adsorbent a charge appropriate for the adsorption of cationic dyes. Unlike carbon materials, mesoporous polymer materials exhibit rather basic properties, which are influenced by the interactions between carbon atoms and the presence of nitrogen.
- The significantly higher affinity of carbons for cationic dyes is mainly related to strong attractive electrostatic forces, while in the polymer-dye system, there are repulsive electrostatic ones. Methylene blue, as a small molecule with a linear shape, is more effectively adsorbed on solids characterized by the presence of micropores and small mesopores. For the adsorption of malachite green and crystal violet, due to their larger particle size and spatially expanded shape, mesoporous materials showing a medium mesopores range are better suited. For adsorbents with small pores, a sieve effect is observed in the applied adsorbates. The mechanism of dye adsorption on carbon and polymer materials is based on electrostatic interactions, π-π interactions (except for systems with P1), free dispersion interactions (London forces), and hydrogen bridges. The higher affinity of the adsorbent for the adsorbate also translates into kinetics; in the case of polymers, crystal violet is adsorbed faster despite the larger size and spatially expanded structure of the molecule. Faster kinetics is observed for carbons—methylene blue systems.

## Figures and Tables

**Table 1:** Reagents and parameters applied in the synthesis of the carbon.

Carbon	Pluronic/Mw ^1^/Formula	Temperature/Process Duration of Silica-Organic Composite Ageing	Temperature/Process Duration of Vacuum Heating of Composite + Catalyst (Stage 1)	Temperature/Process Duration of Vacuum Heating of Composite + Catalyst (Stage 2)	Temperature/Process Duration of Carbonisation
C1	PE9400/4600 g/mol/(EO)_21_(PO)_47_(EO)_21_	70 °C/24 h	100 °C/12 h	160 °C/12 h	800 °C/6 h
C2	PE6400/2900 g/mol /(EO)_13_(PO)_30_(EO)_13_	70 °C/24 h	100 °C/12 h	160 °C/12 h	800 °C/6 h
C3	PE9400/4600 g/mol /(EO)_21_(PO)_47_(EO)_21_	120 °C/24 h	100 °C/12 h	160 °C/12 h	800 °C/6 h
C4	PE6400/2900 g/mol /(EO)_13_(PO)_30_(EO)_13_	120 °C/24 h	100 °C/12 h	160 °C/12 h	800 °C/6 h

^1^ Molecular weight.

**Table 2:** Reagents applied in the synthesis of mesoporous polymer materials.

Polymer	Monomers (g)			Diluents (mL)		Stabilizers (g)	
	VP	TRIM	DVB	Toluene	Dodecane	PVA	CaCl_2_
P1	3.7	11.3	-	22.5	-	6.5	-
P2	6.9	-	8.1	19.1	3.4	6.5	30

**Table 3:** Structure and physicochemical properties of the adsorbates.

Dye Code	Chemical Formula	Molecular Weight [g/mol]	Ionisation Constant, pKa	Water Solubility [%]	D_min_/D_max_^1^ [Å]	Ref.
MB	C_16_H_18_N_3_ClS	319.85	2.6; 11.2	4.36; 4	4.6/9.9	[60]
MG	C_23_H_25_ClN_2_	364.91	6.9	4	8.0/9.7	[60,61]
CV	C_25_H_30_N_3_Cl	407.99	0.8; 9.4	1.7	9.5/9.7	[60]

D_min_/D_max_^1^—the distance between the most remote atoms in a molecule measured using Mercury 3.7 (Build RC1) tools.

**Table 4:** Textural characteristics of carbon and polymer materials.

Carbon	*S_BET_* [m^2^/g]	*S_mic_* [m^2^/g]	*V_t_* [cm^3^/g]	*V_mic_* (t-plot) [cm^3^/g]	*D_h_* [nm]	*D_mo_* (ads. BJH) [nm]	*D_mo_* (des. BJH) [nm]
C1	502	243	0.30	0.11	2.4	3.14	2.75
C2	760	40	0.61	0.01	3.2	3.43	3.24
C3	508	215	0.29	0.10	2.2	2.95	2.58
C4	649	37	0.65	0.01	2.7	4.05	3.74
P1	515	-	1.06	0.08	8.3	9	9.3
P2	515	-	0.75	-	5.8	6.3	6.1

**Table 5:** XPS results for all investigated samples. Positions of general peaks as a signal of photoelectrons from various energetic levels and atomic concentration with standard deviation.

Sample Identifier	Name	Position	FWHM	%At. Conc.	% St.Dev.
C1	C1s	284.5	1.8	84.8	0.105
	O1s	532.8	2.5	11.5	0.081
	Si2p	103.0	2.5	3.7	0.079
C2	C1s	284.5	1.8	88.4	0.087
	O1s	532.8	2.4	9.2	0.066
	Si2p	103.0	2.5	2.4	0.064
C3	C1s	284.5	1.7	82.4	0.156
	O1s	532.8	2.6	13.2	0.114
	Si2p	103.0	2.3	4.4	0.129
C4	C1s	284.5	1.7	91.0	0.153
	O1s	532.8	2.6	7.3	0.126
	Si2p	103.0	2.6	1.7	0.097
P1	C1s	285.0	2.9	76.9	0.119
	N1s	399.0	2.4	3.6	0.090
	O1s	531.8	3.3	19.6	0.098
P2	C1s	285.0	2.5	92.3	0.149
	N1s	399.8	2.5	2.6	0.140
	O1s	531.8	3.1	5.1	0.070

**Table 6:** Parameters of the Generalized Langmuir equation for the dye adsorption on selected activated carbons and polymer materials.

Adsorption System	a_m_	m	n	log K	R^2^
MB (C3)	1.01	0.25	1	1.33	0.98
MB (C4)	0.88	0.26	1	1.62	0.97
MB (P1)	0.038	1	1	1.12	0.84
MB(P2)	0.044	1	1	1.26	0.89
MG (C3)	0.60	0.32	1	0.96	0.97
MG (C4)	0.65	0.26	1	1.19	0.98
CV (C3)	0.33	0.22	1	1.24	0.96
CV (C4)	0.44	0.30	1	1.35	0.99
CV (P1)	0.050	1	1	2.41	0.86
CV (P2)	0.038	1	1	2.26	0.86

## Data Availability

The data are available by the corresponding author.

## Supplementary Materials

The following supporting information can be downloaded at: https://www.mdpi.com/article/10.3390/ma17061374/s1, Figure S1: Distance between the most remote atoms in a molecule of (A) methylene blue, (B) malachite green and (C) crystal violet measured by means of of Mercury 3.7 (Build RC1) tools; Figure S2: Comparison of fitting quality by using the FOE, SOE, MOE, m-exponential and f-MOE equations for a description of kinetic data for the MB (C3) system (A) and the MB (P2) system (B). title; Table S1: Comparison of the parameters of various kinetic equations.
