# Supplementary material for: Mesoporous Carbons and Highly Cross-Linking Polymers for Removal of Cationic Dyes from Aqueous Solutions—Studies on Adsorption Equilibrium and Kinetics
Source: Materials (Basel). 2024 Mar 17;17(6):1374. doi: 10.3390/ma17061374 (PMC10972029; doi:10.3390/ma17061374)
Supplement: Supplementary file 1 [file materials-17-01374-s001.zip › materials-2891363-supplementary material.pdf]

materials

MDPI

Article

Mesoporous Carbons and Highly Cross-linking Polymers for Removal of Cationic Dyes from Aqueous Solutions—Studies on Adsorption Equilibrium and Kinetics

Malgorzata Zienkiewicz-Strzalka, Magdalena Blachnio, Anna Derylo-Marczewska *, Szymon Winter and Malgorzata Maciejewska

Faculty of Chemistry, Maria Curie-Sklodowska University, M. Curie-Sklodowska Sq. 3, 20-031 Lublin, Poland; malgorzata.zienkiewicz-strzalka@mail.umcs.pl (M.Z.-S.); magdalena.blachnio@mail.umcs.pl (M.B.); szymonwinter123@wp.pl (S.W.); mmacieje@umcs.pl (M.M.)

* Correspondence: anna.derylo-marczewska@mail.umcs.pl; Tel.: +48-8153-755-49

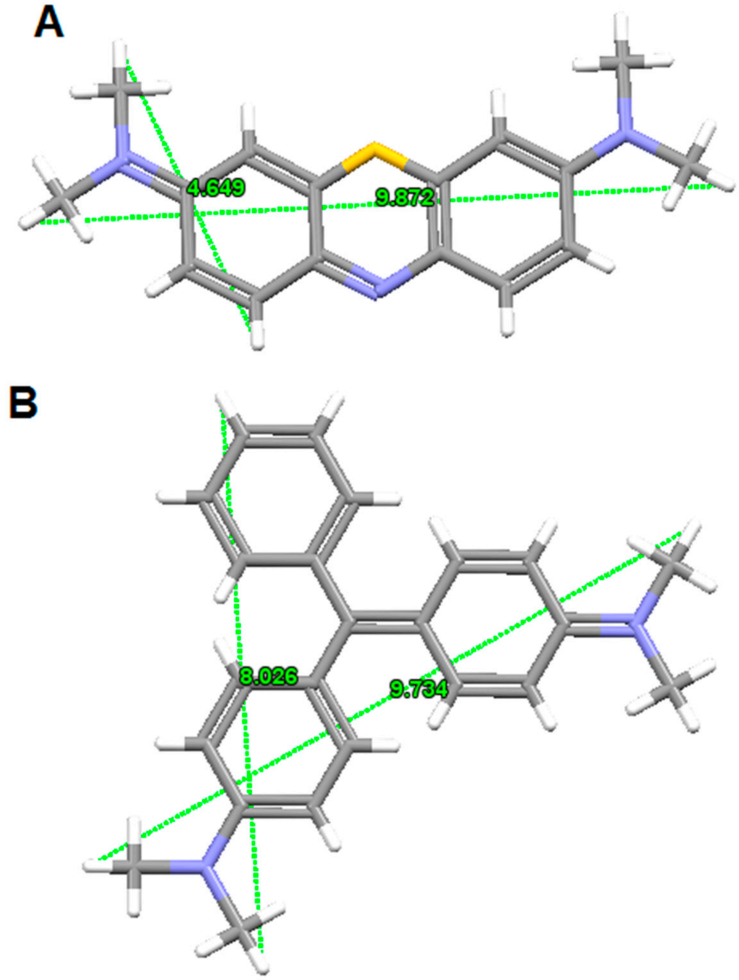

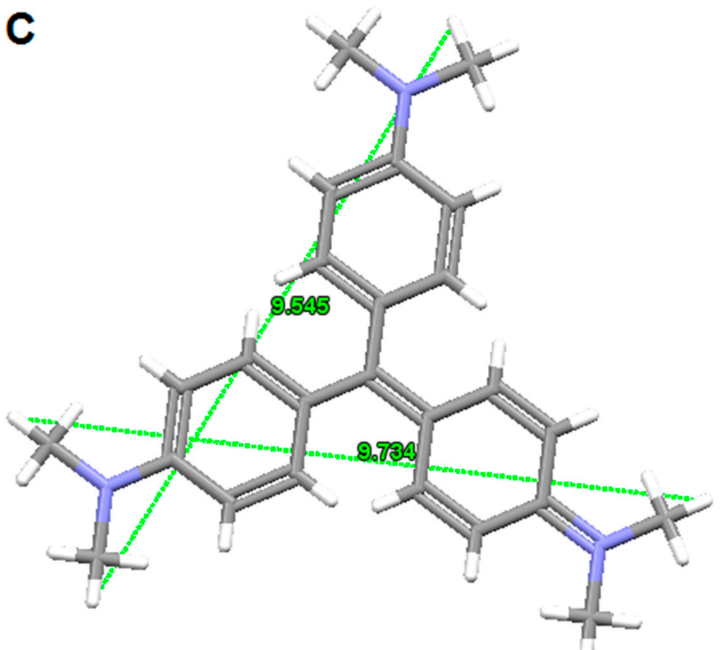

Figure S1. Distance between the most remote atoms in a molecule of (**A**) methylene blue, (**B**) malachite green, and (**C**) crystal violet measured by means of Mercury 3.7 (Build RC1) tools.

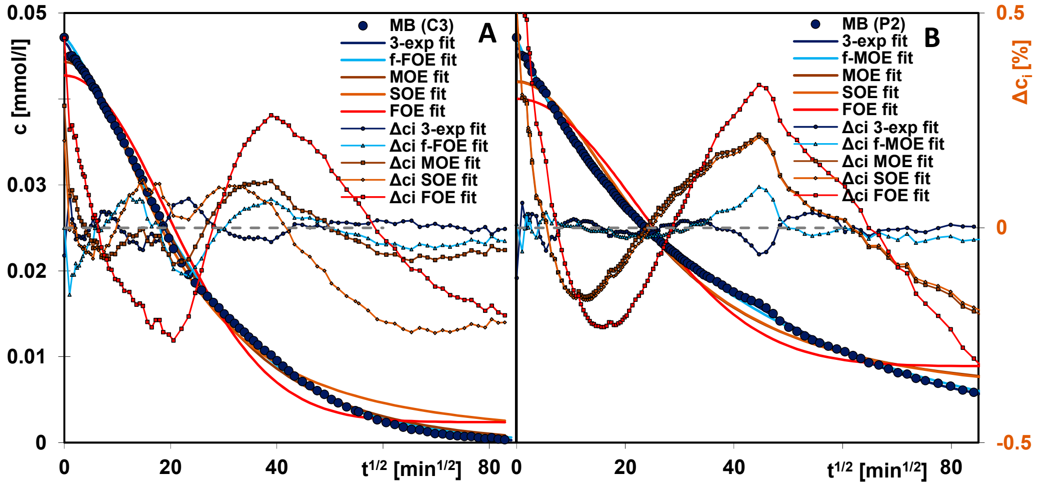

Figure S2. Comparison of fitting quality by using the FOE, SOE, MOE, m-exponential, and f-MOE equations for a description of kinetic data for the MB (C3) system (**A**) and the MB (P2) system (**B**).

Table S1. Comparison of the parameters of various kinetic equations.

Adsorption system	fit	f_2/p	log k [1]	$t_{0.5}$ [min] [2]	u_{eq}	SD(c/c_0) [%]	1-R^2
MB (C3)	FOE	0	-2.87	513	0.94	3.63	$1.09 \cdot 10^{-2}$
	SOE	1	-2.61	412	1	2.75	$6.68 \cdot 10^{-3}$
	MOE	0.84	-3.49	469	1	1.42	$1.6 \cdot 10^{-3}$
	m-exp	--	-2.78	416	1	0.57	$2.53 \cdot 10^{-4}$
	f-FOE	0/0.65	-2.87	420	1	1.12	$1.03 \cdot 10^{-3}$
MB (C4)	FOE	0	-2.24	121	1	1.91	$4.04 \cdot 10^{-3}$
	SOE	1	-1.92	84	1	5.93	$4.75 \cdot 10^{-2}$
	MOE	0.13	-2.28	119	1	1.88	$3.92 \cdot 10^{-4}$

	m-exp	--	-2.16	99	1	0.74	$5.86\cdot10^{-3}$
	f-MOE	-1/0.77	-2.00	114	1	1.57	$2.62\cdot10^{-3}$
MB (P1)	FOE	0	-2.28	133	0.91	3.13	$1.93\cdot10^{-2}$
	SOE	1	-2.05	112	0.97	2.69	$1.41\cdot10^{-2}$
	MOE	0.77	-2.73	113	0.93	2.54	$1.25\cdot10^{-2}$
	m-exp	--	-2.10	87	0.98	0.69	$8.60\cdot10^{-4}$
	f-MOE	-1/0.53	-1.88	91	0.93	2.01	$7.66\cdot10^{-3}$
MB (P2)	FOE	0	-2.99	674	0.81	4.77	$4.12\cdot10^{-2}$
	SOE	1	-2.84	699	0.91	3.03	$1.62\cdot10^{-2}$
	MOE	0.99	-5.20	675	0.90	3.05	$1.63\cdot10^{-2}$
	m-exp	--	-2.91	570	0.92	0.49	$3.88\cdot10^{-4}$
	f-FOE	0/0.47	-3.19	707	1	0.55	$5.10\cdot10^{-4}$
MG (C3)	FOE	0	-3.08	826	0.93	3.91	$1.71\cdot10^{-2}$
	SOE	1	-2.88	764	1	2.61	$7.89\cdot10^{-3}$
	MOE	0.99	-5.21	729	1	2.70	$8.03\cdot10^{-3}$
	m-exp	--	-3.01	710	0.99	0.30	$9.70\cdot10^{-5}$
	f-MOE	-1/0.52	-2.78	726	1	0.62	$4.23\cdot10^{-4}$
MG (C4)	FOE	0	-2.40	173	0.97	3.01	$1.19\cdot10^{-2}$
	SOE	1	-2.11	129	1	3.21	$1.50\cdot10^{-2}$
	MOE	0.75	-2.84	154	1	1.85	$4.49\cdot10^{-3}$
	m-exp	--	-2.22	116	0.99	0.35	$1.57\cdot10^{-4}$
	f-MOE	-1/0.56	-2.04	130	1	0.88	$9.84\cdot10^{-4}$
CV (C3)	FOE	0	-3.30	1379	0.92	3.33	$1.10\cdot10^{-2}$
	SOE	1	-3.12	1309	1	3.06	$9.63\cdot10^{-3}$
	MOE	0.99	-6.02	1279	0.99	3.19	$1.04\cdot10^{-2}$
	m-exp	--	-3.28	1311	0.98	0.32	$9.50\cdot10^{-5}$
	f-MOE	-1/0.58	-3.06	1365	1	0.98	$1.41\cdot10^{-4}$
CV (C4)	FOE	0	-2.81	446	0.89	3.67	$1.81\cdot10^{-2}$
	SOE	1	-2.67	466	1	1.97	$5.31\cdot10^{-3}$
	MOE	0.99	-5.55	454	1	2.05	$5.73\cdot10^{-3}$
	m-exp	--	-2.77	406	1	0.38	$1.84\cdot10^{-4}$
	f-MOE	-0.56/0.54	-2.67	415	1	0.46	$2.80\cdot10^{-4}$
CV (P1)	FOE	0	-2.31	140	0.98	2.70	$8.53\cdot10^{-3}$
	SOE	1	-1.98	95	1	4.13	$2.30\cdot10^{-2}$
	MOE	0.62	-2.59	126	1	2.00	$4.70\cdot10^{-3}$
	m-exp	--	-2.12	92	0.99	0.25	$6.82\cdot10^{-5}$
	f-MOE	-1/0.61	-1.98	111	1	1.27	$1.87\cdot10^{-3}$
CV (P2)	FOE	0	-2.74	383	0.95	3.25	$9.70\cdot10^{-3}$
	SOE	1	-2.51	322	1	2.27	$5.03\cdot10^{-3}$
	MOE	0.86	-3.43	356	1	1.00	$9.16\cdot10^{-4}$

m-exp	--	-2.69	337	0.99	0.38	$1.27 \cdot 10^{-4}$
f-MOE	0.45/0.773	-2.99	333	0.99	0.42	$1.99 \cdot 10^{-4}$

[1] k: k_1-FOE, f-FOE and MOE, f-MOE; k_2-SOE and f-SOE; k_{avg}-m-exp; [2] $t_{0.5}$ for m-exp is the overall half-time calculated numerically from $t_{0.5,i}$ for each of the terms.
